# Maternal depressive symptoms and early childhood temperament before and during the COVID‐19 pandemic in the United Kingdom

**DOI:** 10.1002/icd.2354

**Published:** 2022-06-14

**Authors:** Abigail Fiske, Gaia Scerif, Karla Holmboe

**Affiliations:** ^1^ Department of Experimental Psychology University of Oxford Oxford UK; ^2^ School of Psychological Science University of Bristol Bristol UK

**Keywords:** COVID‐19 pandemic, infants, maternal depressive symptoms, temperament, young children

## Abstract

The COVID‐19 pandemic is an unexpected and major global event, with the potential to have many and varied impacts on child development. However, the implications of the pandemic for maternal depressive symptoms, early childhood temperament dimensions, and their associations, remain largely unknown. To investigate this, questionnaires were completed by mothers (*N* = 175) before and during the pandemic when their child was 10‐ and 16‐months old (Study 1), and by an extended group of mothers with young children (6–48 months; 66 additional mothers) during the first and second national lockdowns in the United Kingdom in 2020 (Study 2). Results indicated that while maternal pandemic‐related stress decreased over the first 6 months of the pandemic, there was an increase in mothers who reported feeling some level of pandemic‐specific depression. Despite this, we did not observe an increase in the severity of global maternal depressive symptoms, or any negative impact of the pandemic on the development of temperament in infancy and early childhood.

**Highlights**

- This exploratory study used longitudinal questionnaire data to investigate the potential impact of the COVID‐19 pandemic on maternal depressive symptoms and early childhood temperament.
- We observed associations between maternal depressive symptoms and child temperament both before and during the pandemic.
- However, there was no evidence of an increase in global maternal depressive symptoms, nor any negative impact of the pandemic on early temperament development.

## 1 INTRODUCTION

The COVID‐19 pandemic has, in many ways, fundamentally changed the environment in which infants and young children are developing. Families with young children have faced unpredictable and uncertain futures and many have experienced prolonged periods of isolation from their support networks. Since increases in maternal depressive symptoms (MDS) during the pandemic are likely to be a widespread, global problem (Brown et al., 2020; Racine et al., 2021; Thapa et al., 2020), it is critically important that we understand, as early as possible, the impact this may have on aspects of development in early childhood. The current exploratory study investigated the changes in, and longitudinal associations between, MDS and early childhood temperament before and during the COVID‐19 pandemic in the United Kingdom (UK).

Infancy and early childhood mark an important period for the development of temperament; a psychobiological construct that refers to individual differences in dispositional traits such as emotional reactivity and behavioural regulation (Putnam et al., 2001; Rothbart & Derryberry, 1981). Three broad temperament dimensions are often studied within the early childhood literature. Surgency, which refers to the frequent expression of positive emotions and a high activity level, Negative Affect, a trait characterized by expressions of frustration, anger, fear and sadness, and Effortful Control or Orienting/Regulatory Capacity, the ability to exert attentional control over behaviour and emotions (Gartstein & Rothbart, 2003; Putnam et al., 2001; Rothbart et al., 2003). Orienting/Regulatory Capacity, which is measured in infancy, is predictive of Effortful Control in early childhood (Putnam et al., 2006, 2014). These temperament characteristics can be observed in rudimentary forms from early in infancy (Gartstein & Rothbart, 2003; Putnam et al., 2001; Rothbart, 2011) and research has demonstrated both homotypic and heterotypic longitudinal associations between temperament in infancy and later in childhood (Holmboe, 2016; Komsi et al., 2006; Putnam et al., 2008).

Individual differences in ratings of early temperament (Carranza et al., 2013; Putnam et al., 2006, 2008; Rothbart et al., 2000), MDS (Rigato, Stets, et al., 2020; Takács et al., 2019) and maternal stress (Crnic et al., 2005) have been shown to remain stable over time. However, we know that temperament development is subject to change under the influence of factors such as changes in parent–child interaction, including those caused by parental stress (Repetti & Wood, 1997) and differences in attachment security (Belsky et al., 1991). Since individual differences in early temperament dimensions have been associated with many later childhood outcomes, including emotional and behavioural difficulties (Abulizi et al., 2017), conduct problems (Rigato, Charalambous, et al., 2020) and academic skills (Blair & Razza, 2007), and also adult outcomes such as anxiety during the COVID‐19 pandemic (Zeytinoglu et al., 2021), it is important to investigate factors that may influence temperament development before and during the pandemic.

One such factor is clearly maternal mental health. Multiple studies have highlighted the robust association between maternal mental health and aspects of temperament across infancy and early childhood (Bates et al., 2020; Feldman et al., 2009; Spry et al., 2020). Outside of a pandemic context, maternal stress was found to be both concurrently and longitudinally associated with higher levels of child Negative Affect (Pesonen et al., 2008). This study also found evidence of bi‐directional associations between maternal stress and infant temperament in infancy (6 months) and early childhood (5½ years; Pesonen et al., 2008). Further, research has found significant cross‐sectional and longitudinal associations between MDS and infant Negative Affect. For example, Rigato, Stets, et al. (2020) found that MDS predicted infant Negative Affect from as early as 2 weeks after birth and across the first 6 months of life. The authors also found that depressed mothers had difficulty coping with parenting and expressed a negative perception of their relationship with their child (Rigato, Stets, et al., 2020). Similarly, it has been suggested that common characteristics of depressed mothers such as a lack of responsiveness and less positive engagement with the child may contribute to the child's expression of Negative Affect (Campbell et al., 1995; Cummings & Davies, 1994; Rigato, Stets, et al., 2020). Infant‐driven effects have also been found, whereby characteristics associated with high levels of infant Negative Affect (e.g., increased distress or irritability that is difficult to soothe) were linked to a heightened risk of MDS later in infancy (Cutrona & Troutman, 1986; Murray et al., 1996). Thus, the direction of the association between MDS and child Negative Affect remains unclear.

Associations between MDS and other temperament dimensions have also been found. In infancy, increased MDS were associated with a decrease in Orienting/Regulatory Capacity from 8 to 10 months of age (Gartstein & Hancock, 2019), and in early childhood, higher levels of MDS were associated with lower Effortful Control (Gartstein et al., 2013). Further research has shown that a combination of high MDS and low child Effortful Control increased the likelihood of the child exhibiting externalizing behavioural difficulties (Gartstein & Fagot, 2003). The authors of this study proposed that depressive symptoms may impact the attitudes, parenting strategies and emotional availability of the mother (Gartstein & Fagot, 2003). For example, a depressed mother with low confidence in her parenting abilities and high levels of negative emotion may be less likely to provide the child with effective strategies and opportunities to practice regulating their own behaviour and emotions. The impact of MDS on child Surgency is less well‐studied, although Gartstein and Hancock (2019) found that higher levels of MDS contributed to increases in approach behaviours (a common Surgency characteristic) of infants from 6 to 8 months of age. It was suggested that this may be a compensatory mechanism in response to the depressed mothers' lack of responsiveness or withdrawal (Gartstein & Hancock, 2019; Pelaez et al., 2007). Overall, this body of evidence highlights the important links between the mother's mood and her child's temperament.

While the full picture of the influences of the pandemic on maternal mental health is still incomplete, new research is beginning to suggest an increase in anxiety and depressive symptoms in pregnant women (Filippetti et al., 2022; Lebel et al., 2020; Morris et al., 2021; Wu et al., 2020) and mothers of young and school‐age children (Cameron et al., 2020; Racine et al., 2021). However, an early meta‐analysis suggests that maternal anxiety may be more impacted by the pandemic than depressive symptoms (Hessami et al., 2020). These findings are concerning since even in the absence of a pandemic, MDS have significant associations with child self‐regulatory (Rigato, Stets, et al., 2020; Shapiro et al., 2018), cognitive (Kingston et al., 2012) and neural (Porto et al., 2020) development. Indeed, there is considerable evidence that demonstrates the sustained impact of MDS on later childhood emotional and behavioural difficulties (see Goodman et al., 2020, for meta‐analysis), even when symptom severity is low (Cents et al., 2013; Giallo et al., 2015). As such, it is likely that a potential increase in MDS as a result of the COVID‐19 pandemic would have adverse consequences for key aspects of children's development (Burke, 2003; Karam et al., 2016; Talge et al., 2007), including early temperament (Rigato, Stets, et al., 2020). Therefore, due to the recency of the pandemic and the importance of temperamental dimensions as predictors of later outcomes, it is necessary to conduct research that probes the potential impact of pandemic‐related stress on MDS and the development of temperament during the first years of life.

### 1.1 The current study

**FIGURE 1:**
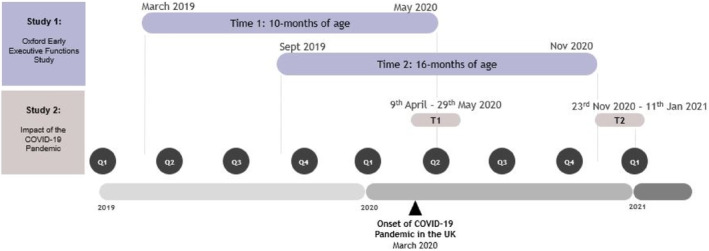
Timeline of data collection. Calendar years have been divided into quarters (e.g., Q1 which refers to the first quarter of the year; January–March) and are plotted on the *x*‐axis. Individual data points in Study 1 were collected at *different points in time* over the course of a year, but children were all the same age when data were collected (10‐ and 16‐months). Study 2 data were collected in response to the COVID‐19 pandemic. This data were collected at the *same points in time* (first and second national lockdowns) and so the age of children at each data collection point varied. T1 refers to Time 1 which took place during the first national lockdown in April 2020, and T2 refers to Time 2 which took place during the second national lockdown in November 2020

The overarching aim of this study is to assess the impact of the COVID‐19 pandemic on MDS, child temperament, and their associations across the early childhood period. The study was largely exploratory, as the pandemic was an unprecedented global event with the potential to have a wide range of impacts on child development. Using questionnaire data from two overlapping samples of mothers collected before and during the pandemic (see “Materials and Methods” for further information about our samples and data collection timelines, and see also Figure 1), we ask a number of questions to probe this: First, is the expected longitudinal stability in ratings of MDS and temperament still observed during the pandemic? Second, do ratings of MDS increase over time as a result of the pandemic as has been shown in other studies, and, if so, what are the implications of this for early temperament development? Third, are the consistently demonstrated concurrent and longitudinal associations between MDS and dimensions of early childhood temperament altered as a result of the COVID‐19 pandemic?

### 1.2 Aims and hypotheses

By comparing pre‐ and during‐pandemic data collected across the same developmental period of 10‐ to 16‐months of age (Study 1), we aimed to investigate whether MDS and the typical development of temperament in infancy was impacted by the onset of the pandemic. In data collected before the pandemic, we expected to observe longitudinal stability in ratings of MDS and overall temperament indices of Negative Affect, Effortful Control and Surgency (Putnam et al., 2006, 2008; Rigato, Stets, et al., 2020; Takács et al., 2019). We also expected to find concurrent and longitudinal associations between MDS and specific dimensions of infant temperament, such as Negative Affect (Bates et al., 2020; Feldman et al., 2009; Granat et al., 2017; Rigato, Stets, et al., 2020), Effortful Control (Choe et al., 2014; Gartstein & Fagot, 2003; Rigato, Charalambous, et al., 2020), and Surgency (Gartstein & Hancock, 2019).

Based on the emerging literature on the effect of the COVID‐19 pandemic on maternal mental health (Filippetti et al., 2022; Lebel et al., 2020; Morris et al., 2021; Racine et al., 2021; Wu et al., 2020), we predicted an increase in MDS from before to during the pandemic (Study 1) and across the pandemic assessment points (Study 2). Given that higher levels of MDS have previously been associated with poorer child self‐regulation (Rigato, Charalambous, et al., 2020; Rigato, Stets, et al., 2020), we also anticipated an increase in child Negative Affect and a decrease in Effortful Control during the pandemic. We had no specific hypotheses about if, or how, Surgency ratings would change. In Study 2, we additionally measured maternal stress related specifically to the pandemic and expected an increase across pandemic assessment points, potentially contributing to mothers' self‐reported depressive symptoms.

### 1.3 Participant samples and data collection timelines

#### 1.3.1 Study 1

We collected MDS and infant temperament questionnaire data from a sample of mothers (*N* = 175) at two time points (Time 1: when their child was 10‐months of age, Time 2: when their child was 16‐months of age) as part of an ongoing longitudinal study (the Oxford Early Executive Functions [EF] study). The Oxford Early EF study aimed to collect parent‐report questionnaire and child behavioural data from the same cohort of participants at several age points across infancy and early childhood: 10‐months (Time 1), 16‐months (Time 2), 24‐months (Time 3) and 30‐months (Time 4). Data collected at Time 3 and 4 are not reported in the current study. Data collection for the Oxford Early EF study began in April 2019 and participants were continuously recruited into the study at Time 1 (when the child turned 10‐months of age) until April 2020; see Figure 1 for illustration of data collection timelines. Note that because of this continuous study recruitment, children did not reach the age milestones at the same point in time.

The onset of the pandemic in March 2020 meant that in‐person data collection could not continue; however, questionnaire data were still collected from parents at each time point as their child reached the relevant age milestone. Since the data collection period for individual children reaching Time 1 (10‐months) and Time 2 (16‐months) spanned the onset of the pandemic and extended into the first 8 months of the pandemic (see Figure 1), part of this data was collected entirely before the pandemic. Therefore, for participants who turned 16‐months *before* the pandemic onset (March 2020), both Time 1 (10‐months) and Time 2 (16‐months) occurred before the pandemic. As such, data reflect the child's temperament and the mother's depressive symptoms without any pandemic influence. Data collected from participants in this group are described as “pre‐pandemic” data. Another part of the data was collected across the pandemic onset, in that, data from Time 1 (10‐months of age) was collected before the pandemic onset, whereas the data from Time 2 (16‐months) was collected after the onset (i.e., during the pandemic). This means that only the 16‐month data (Time 2) will reflect child temperament and MDS with potential pandemic influence. As such, data collected from participants in this group are described as “pre‐to‐during pandemic” data.

#### 1.3.2 Study 2

Here we aimed to investigate how changes in MDS and maternal stress related specifically to the pandemic would impact the development of temperament across infancy and early childhood. Data for Study 2 were collected during the first and second national lockdowns in the UK in 2020—an overview of the national measures and restrictions in place during this period is provided in Supporting Information 1. We collected data using the same depression and temperament questionnaires (as in Study 1) from a large sample of mothers with infants and young children spanning a broad age range (*N* = 220, age range 6 to 48 months), in contrast to the discrete ages for Study 1 (whose data were collected when infants reached 10‐ and 16‐months of age). The sample for Study 2 overlapped with that in Study 1 (described above) as mothers participating in the Oxford Early EF study were recruited to contribute data also during the pandemic. Therefore, of the 220 participants who contributed data to Study 2, 154 had participated in Study 1 and 66 participants were recruited from a pool of participants who had engaged in pilot test sessions for the Oxford Early EF study but had not previously contributed questionnaire data.

## 2 MATERIALS AND METHODS: STUDY 1

### 2.1 Participants

Participants were drawn from a sample of 187 mothers of infants (101 male) who were taking part in a longitudinal study investigating early executive function development (the Oxford Early Executive Functions [Oxford Early EF] study). The Oxford Early EF study received full ethical approval from the University of Oxford Central University Research Ethics Committee: R57972/RE010. All 187 mothers were approached to complete questionnaires, but 6 participants (3 male children) withdrew from the study before any data was collected. As such, questionnaires were sent to 181 mothers at Time 1 (10‐months of age). Due to attrition between assessment points, questionnaires were sent to 168 of these mothers at Time 2 (16‐months of age); see Supporting Information 2.1 for further details.

As part of the Oxford Early EF study, mothers completed maternal depression and infant temperament questionnaires when their child was 10‐ (Time 1) and 16‐months (Time 2) of age. Our final sample comprised 175 mothers (93 male children) who contributed a response (partial or complete) at at least one assessment point. Infants in the reported sample (*N* = 175) were primarily from families of high to middle socioeconomic status, as indicated by the index of multiple deprivation decile (*M* = 7.47, SD = 2.2), annual household income (*M* = £76,343, SD = £31,565), and the mother's years in education (*M* = 17.91 years, SD = 3.10 years). The ethnicity of this sample (70% White British) is reflective of the ethnic demographic of the city in which this research was conducted, according to the 2011 UK Census. See Supporting Information 3.1 for further demographic information about the reported sample.

#### 2.1.1 Missing data

Full responses at both 10‐ and 16‐months were received from 118 participants (61 male children). See Table 1 for questionnaire response rates, and see also Supporting Information 2.2 for comprehensive details of the missing data. According to the results of Little's missing completely at random test (Little, 1988) conducted in SPSS, the data in this study were missing completely at random; *χ* ^2^ (34) = 33.029, *p* = 0.515. See Supporting Information 2.2 for comprehensive information about missing data in this study including a missing data pattern analysis, and see also the Section 3.4 for how missing data were handled in our analyses.

**TABLE 1:** Questionnaire response rate by assessment point

	Time 1: 10‐months	Time 2: 16‐months
*N* of questionnaires sent	181	168
*N* of complete responses received	156	129
Complete response rate	86.2%	73.3%
*N* of partial responses received	17	15
Total *N* of responses received	173	144
Total response rate (partial or complete)	95.6%	85.7%

*Note*: Partial Responses refer to the receipt of questionnaire packs in which some, but not all questionnaires had been completed. Further information regarding the reasons why questionnaires were not sent to some participants at each assessment point are detailed in Supporting Information 2.1.

#### 2.1.2 Data exclusions

The inclusion criteria for the Oxford Early EF study required that infants were born at full‐term (at least 36 weeks) and/or with a minimum birth weight of 2500 g/5.5 pounds. Infants who experienced birth complications leading to health‐related concerns were excluded from all analyses (*N* = 3, male). Data from questionnaires that were received outside of the assessment windows (9–12 months of age at Time 1 and 16–18 months of age at Time 2) were not included in analyses (*N* = 2 females, 16‐month temperament questionnaire). Data from the depression questionnaire were excluded if the questionnaire was completed by the child's father (*N* = 2, females, 16‐month assessment point). See Section 3.4 for how missing data were handled in our statistical analyses.

### 2.2 Materials

#### 2.2.1 Maternal depressive symptoms

The Beck Depression Inventory, second edition (BDI‐II; Beck et al., 1996) was completed by mothers as a measure of their depressive symptoms. This scale consists of 21 items that are rated from 0 to 3, although two items include additional options to reflect direction of change (e.g., increase or decrease in appetite). The score for each question is summed to generate an overall BDI‐II score. According to a comprehensive review of the psychometric properties of the BDI‐II (Wang & Gorenstein, 2013), the average internal consistency (Cronbach's alpha) from 118 studies is excellent; *α* = 0.90 (range: *α* = 0.83–0.96). In this study, internal consistency was also excellent at both Time 1: 10‐months (*α* = 0.93) and Time 2: 16‐months (*α* = 0.94).

#### 2.2.2 Infant temperament

The 37‐item Infant Behaviour Questionnaire‐Revised, Very Short Form (IBQ‐R‐VSF, Putnam et al., 2014) contains questions that relate to the infant's behaviour in the last week and are associated with three temperament dimensions: Surgency, Negative Affect and Orienting/Regulatory Capacity. Items are rated on an 8‐point scale from *Never* (1) to *Always* (7), including *Does Not Apply* (0, excluded from analysis). Some items are reverse scored according to a standard scoring system (Putnam et al., 2014). A mean score is calculated for each temperament dimension: the higher the score, the more of that trait the child displays. Putnam et al. (2014) reported the average internal consistency (Cronbach's alpha) from six separate studies for each of the three scales derived from the IBQ‐R‐VSF; *α* = 0.75 (Orienting/Regulatory Capacity), *α* = 0.77 (Surgency) and *α* = 0.78 (Negative Affect). In the current study, Cronbach's alpha for each of the three scales ranged from 0.48 to 0.75 across both ages, see Supporting Information 4.1 for full details. Since the Cronbach's alpha for the IBQ‐R‐VSF Surgency scale was too low to be considered reliable (*α* = 0.48 at 10 months, *α* = 0.52 at 16 months), we do not include this scale in our reported analyses. However, results of analyses of the Surgency scale can be found in Supporting Information 5.1. To maintain consistent wording of temperament constructs throughout the article, we will use the term “Effortful Control” when referring to the Orienting/Regulatory Capacity scale in the IBQ‐R‐VSF.

### 2.3 Procedure

Mothers completed the BDI‐II (depressive symptoms) and IBQ‐R‐VSF (infant temperament) questionnaires online via Qualtrics at two different time points: Time 1) when their child was 10‐months of age, and Time 2) when their child was 16‐months of age. Before the pandemic, a link to the temperament questionnaire was sent via email to mothers approximately 1 week before their scheduled visit to the Oxford University Babylab. The link to the depressive symptoms questionnaire was sent immediately after the visit. When testing sessions were cancelled due to pandemic‐related restrictions (from March 2020), mothers were sent links to both questionnaires approximately 1 week before their child's 10‐ and 16‐month “birthday”. Responding participants (*N* = 175) were organized into sub‐samples based on whether data were received before or during the pandemic (Table 2). Data were considered as being collected pre‐pandemic if received on or before 23rd March 2020; the date that the first UK lockdown was announced.

**TABLE 2:** Participant sub‐samples

Sub‐sample	Total *N* responses	Details
Pre‐pandemic	84	All data collected before the pandemic. This sub‐sample acts as a control group for which changes in MDS and infant temperament from 10‐months to 16‐months can be assessed in the absence of a pandemic
Pre‐ to during pandemic	70	Provided 10‐month data *before* and 16‐month data *during* the pandemic. This sub‐sample enables the examination of how changes in MDS and infant temperament ratings across an equivalent 6 month period (10‐ to 16‐months) are potentially influenced by the COVID‐19 pandemic. The length of pandemic exposure (days) from the start of the first national lockdown to receipt of the 16‐month questionnaires ranged from 8 to 185 days (see Supporting Information 6.1)
During pandemic	21	Contributed both 10‐ and 16‐month data *after* the pandemic onset. Nine of these participants provided complete data at both assessment points. For analysis purposes, this sample (*N* = 21) is included in the “pre‐ to during pandemic” group. The length of pandemic exposure (days) ranged from 6 to 144 days for the 10‐month questionnaires, and 162 to 246 days for the 16‐month questionnaire (see Supporting Information 6.1). To test for convergence in results (which was found) we also conducted analyses without this group included in the “pre‐ to during pandemic” group; reported in Supporting Information 7

*Note*: The full sample of participants (*N* = 175) were divided into three sub‐samples based on whether their data were collected entirely before the pandemic, in part before and in part during the pandemic or entirely during the pandemic.

### 2.4 Analysis approach

All analyses were conducted in SPSS version 27 and a two‐tailed significance level of *p* < 0.05 was used. Note that since the Cronbach's alpha for the IBQ‐R‐VSF Surgency scale was too low to be considered reliable (*α* = 0.48 at 10 months, *α* = 0.52 at 16 months), we do not include this scale in our reported analyses (results for this scale are reported in Supporting Information 5).

Pearson's correlational analyses were performed to investigate the longitudinal stability of individual differences, and also to examine associations between MDS and temperament variables both within‐assessment points and longitudinally. Confidence intervals (95%) were calculated using bootstrapping (1000 samples). When correlational analyses were conducted, no correction, estimation or imputation was carried out. Instead, data were excluded from analyses via pairwise deletion if data for one of the variables in the correlation were missing for a specific participant.

To test whether ratings of MDS and temperament significantly differed across time points (10‐ to 16‐months), we conducted fully factorial linear mixed models (assuming a diagonal covariance structure) which enabled us to consider all available data instead of excluding participants without complete data at both assessment time points, see Table 1 and also Supporting Information 2.2 for comprehensive details of the missing data in this study. Here, full information maximum likelihood estimation was used to account for missing data.

Separately for each of the three dependent variables (MDS, Negative Affect, and Effortful Control), a linear mixed model with assessment time point (2 levels) and sub‐sample (2 levels) as fixed factors and participants as a random factor were conducted to look at mean changes. These models tested the main effects of assessment time point and sub‐sample, and the assessment time point × sub‐sample interaction (Model 1). Further linear mixed models (Negative Affect and Effortful Control as separate dependent variables) with MDS as a fixed covariate was also conducted (Model 2). A similar model was conducted with pandemic exposure (days) as a fixed covariate and Effortful Control as the dependent variable (Model 3).

## 3 RESULTS

### 3.1 MDS and temperament from 10‐ to 16‐months of age

The final sample consisted of 175 mothers who contributed a response (partial or complete) to at least one assessment point. Descriptive statistics for ratings of MDS and infant temperament at each assessment time point are presented in Table 3 below.

**TABLE 3:** Descriptive statistics for ratings of maternal depressive symptoms and infant temperament

	*N*	Maternal depressive symptoms	*N*	Negative affect	Effortful control
Pre‐pandemic					
Time 1: 10‐months	72	10.64 (9.31)	78	4.22 (0.95)	4.70 (0.69)
Time 2: 16‐months	62	9.90 (10.12)	71	4.28 (0.75)	5.01 (0.64)
Pre‐ to during pandemic					
Time 1: 10‐months	81	9.54 (9.00)	89	4.13 (0.88)	4.84 (0.67)
Time 2: 16‐months	61	8.57 (8.77)	67	4.12 (0.84)	5.16 (0.60)

*Note*: Data in table represent mean, brackets contain standard deviation. *N* represents the number of questionnaire responses that contribute to each statistic.

### 3.2 Longitudinal stability

Results of Pearson's correlation analyses (Table 4) indicated moderate to strong longitudinal stability of individual differences in MDS and the infant temperament dimensions from 10‐ to 16‐months of age in both sub‐samples. All correlations remained significant when controlling the false discovery rate (Benjamini & Hochberg, 1995).

**TABLE 4:** Longitudinal and within‐age associations between ratings of MDS and infant temperament from 10‐ to 16‐months of age

	10‐month MDS	10‐month NA	10‐month EC	16‐month MDS	16‐month NA	16‐month EC
10‐month Maternal Depressive Symptoms (MDS)	–	0.353***, ^a^ [0.183, 0.527] *81*	−0.046, [−0.227, 0.121] *81*	0.861***, ^a^ [0.661, 0.948] *57*	−0.001 [−0.202, 0.224] *62*	0.129 [−0.148, 0.328] *62*
10‐month Negative Affect (NA)	0.170 [−0.037, 0.345] *72*	–		0.213 [0.015, 0.397] *60*	0.338*, ^a^ [0.109, 0.549] *66*	
10‐month Effortful Control (EC)	−0.216 [−0.375, −0.065] *72*		–	−0.132 [−0.323, 0.049] *60*		0.633***, ^a^ [0.469, 0.766] *66*
16‐month Maternal Depressive Symptoms (MDS)	0.872***, ^a^ [0.734, 0.937] *55*	−0.041 [−285, 193] *58*	−0.244 [−0.452, −0.069] *58*	–	−0.133 [−0.359, 0.101] *60*	−0.012 [−0.332, 0.248] *60*
16‐month Negative Affect (NA)	0.094 [−0.160, 0.363] *62*	0.376**, ^a^ [0.170, 0.558] *65*		−0.133 [−0.385, 0.192] *62*	–	
16‐month Effortful Control (EC)	−0.151 [−0.345, 0.064] *62*		0.506***, ^a^ [0.250, 0.685] *65*	−0.080 [−0.279, 0.136] *62*		–

*Note*: Correlations for the pre‐pandemic group are to the left of and below the diagonal (shaded), the pre‐to‐during group are to the right of and above the diagonal (not shaded). Cells show the correlation coefficient, 95% confidence intervals in square brackets and *N* in italics. Blank cells indicate correlations that were not run. Note that pairwise deletion was used such that only participants who contributed data to both variables in the correlation pair were included in analyses.

^a^ Remained significant following the procedure for controlling the false discovery rate.

* *p* < 0.05;

** *p* < 0.01;

*** *p* < 0.001.

### 3.3 Within‐age and longitudinal associations between MDS and temperament

Within‐age correlation analyses (Table 4) revealed a small‐to‐moderate association between MDS and Negative Affect at 10‐months in the pre‐ to during pandemic group, which remained significant when controlling the false discovery rate. No other significant within‐age correlations were found.

Since there was no significant difference between the correlation coefficients of MDS and Negative Affect at 10‐months in the pre‐pandemic group (*r* = 0.170) and the pre‐ to during pandemic group (*r* = 0.353); *Z* = −1.19, *p* = 0.233, and all 10‐month data were collected under the same conditions before the pandemic, the 10‐month data were combined to further examine the association between MDS and Negative Affect. When pooling the 10‐month pre‐pandemic data (which did not include the small sample of 10‐month data collected during the pandemic), there was still a significant association between MDS and Negative Affect, although with a reduced effect size; *r* (132) = 0.257, *p* = 0.003, [*CI* = 0.121, 0.397].

Further correlational analyses were conducted to investigate longitudinal associations between MDS and infant temperament; however, no significant effects were found in either group (Table 4).

### 3.4 Change in ratings from 10‐ to 16‐months of age (by sub‐sample)

#### 3.4.1 Model 1: Change in MDS and temperament from 10‐ to 16‐months of age

In Table 5, we report the estimated marginal means and results of Type III fixed effect tests for models that included assessment point and sub‐sample as fixed factors, and participants as the random factor (Model 1). There was no significant main effect of assessment point, sub‐sample or interaction in the models where MDS or Negative Affect was the dependent variable. However, a significant main effect of assessment point was found when Effortful Control was entered into the model as the dependent variable, which indicated that ratings of Effortful Control were significantly higher at the 16‐month assessment point (*M* = 5.08, SE = 0.051) than at the 10‐month assessment point (*M* = 4.77, SE = 0.053).

**TABLE 5:** Model 1: Type III fixed effects and estimated marginal means

	MDS	Negative affect	Effortful control
Assessment point	*F* (1, 116.342) = 2.441, *p* = 0.121, ${\mathrm{\eta p}}^{2}$ = 0.021	*F* (1, 153.235) = 0.210, *p* = 0.648, ${\mathrm{\eta p}}^{2}$ < 0.001	***F* (1, 143.610) = 37.124, *p* < 0.001,** ${\mathrm{\eta p}}^{2}$ **= 0.205**
Sub‐sample	*F* (1, 164.434) = 1.145, *p* = 0.286, ${\mathrm{\eta p}}^{2}$ = 0.007	*F* (1, 168.078) = 1.530, *p* = 0.218, ${\mathrm{\eta p}}^{2}$ = 0.009	*F* (1, 168.337) = 2.161, *p* = 0.143, ${\mathrm{\eta p}}^{2}$ = 0.013
Assessment point × Sub‐sample	*F* (1, 116.342) = 1.694, *p* = 0.196, ${\mathrm{\eta p}}^{2}$ = 0.014	*F* (1, 153.235) = 0.037, *p* = 0.847, ${\mathrm{\eta p}}^{2}$ < 0.001	*F* (1, 143.610) = 0.158, *p* = 0.692, ${\mathrm{\eta p}}^{2}$ = 0.001
Assessment point			
10‐months	10.03 (0.719)	4.18 (0.070)	4.77 (0.053)
16‐months	9.40 (0.763)	4.22 (0.066)	5.08 (0.051)
Mean difference	0.694 (0.444)	0.037 (0.080)	0.306 (0.050)
Sub‐sample			
Pre‐pandemic	10.44 (1.02)	4.27 (0.079)	4.86 (0.065)
Pre‐ to during pandemic	8.93 (0.981)	4.13 (0.077)	4.99 (0.063)
Mean difference	1.514 (1.42)	0.137 (0.111)	0.132 (0.090)

*Note*: Mean (standard error) are reported. Note that maximum likelihood estimation was used to account for missing data, so all participants who contributed at least some data at either assessment point were included in these analyses.

Bold text indicates significant effects (*p* < .05).

#### 3.4.2 Model 2: Influence of MDS on temperament change from 10‐ to 16‐months

Table 6 contains the estimated marginal means and results of Type III fixed effect tests for the models that included assessment point and sub‐sample as fixed factors, participants as the random factor, and in which MDS was entered as a fixed covariate (Model 2).

**TABLE 6:** Model 2 (MDS as fixed covariate): Estimated marginal means and type III fixed effects

	Negative affect	Effortful control
Assessment point	***F* (1, 148.775) = 9.073,** ***p* = 0.003,** ${\mathrm{\eta p}}^{2}$ **= 0.057**	***F* (1, 135.678) = 5.511,** ***p* = 0.020,** ${\mathrm{\eta p}}^{2}$ **= 0.039**
MDS	*F* (1, 195.680) = 1.329, *p* = 0.250, ${\mathrm{\eta p}}^{2}$ = 0.007	*F* (1, 215.875) = 0.828, *p* = 364, ${\mathrm{\eta p}}^{2}$ = 0.004
Assessment point × MDS	***F* (1, 151.951) = 11.915,** ***p* = 0.001,** ${\mathrm{\eta p}}^{2}$ **= 0.073**	*F* (1, 138.836) = 2.823, *p* = 0.095, ${\mathrm{\eta p}}^{2}$ = 0.020
Sub‐sample	*F* (1, 178.073) = 1.398, *p* = 0.239, ${\mathrm{\eta p}}^{2}$ = 0.008	*F* (1, 186.476) = 0.655, *p* = 0.419, ${\mathrm{\eta p}}^{2}$ = 0.004
Assessment point × Sub‐sample	*F* (1, 148.775) = 0.939, *p* = 0.334, ${\mathrm{\eta p}}^{2}$ = 0.006	*F* (1, 135.678) = 0.674, *p* = 0.413, ${\mathrm{\eta p}}^{2}$ = 0.005
Sub‐sample × MDS	*F* (1, 195.680) = 0.290, *p* = 0.591, ${\mathrm{\eta p}}^{2}$ = 0.001	*F* (1, 215.875) = 0.123, *p* = 0.726, ${\mathrm{\eta p}}^{2}$ < 0.001
Assessment point × Sub‐sample × MDS	*F* (1, 151.951) = 1.442, *p* = 0.232, ${\mathrm{\eta p}}^{2}$ = 0.009	*F* (1, 136.836) = 0.078, *p* = 0.780, ${\mathrm{\eta p}}^{2}$ < 0.001
Assessment point		
10‐months	4.15 (0.071)	4.76 (0.053)
16‐months	4.21 (0.070)	5.04 (0.053)
Mean difference	0.060 (0.084)	0.283 (0.054)
Sub‐sample		
Pre‐pandemic	4.24 (0.081)	4.84 (0.066)
Pre‐ to during pandemic	4.11 (0.079)	4.97 (0.064)
Mean difference	0.128 (0.113)	0.135 (0.091)

*Note*: Mean (standard error) are reported. MDS covariate is entered into the model as 9.6957. Note that maximum likelihood estimation was used to account for missing data in this model, so all participants who contributed at least some data at either assessment point were included in these analyses.

Bold text indicates significant effects (*p* < .05).

When Negative Affect was entered into the model as the dependent variable, we found a significant assessment point × MDS interaction; *F* (1, 151.951) = 11.915, *p* = 0.001, ${\mathrm{\eta p}}^{2}$ = 0.073. Post‐hoc correlational analyses (sub‐samples combined) confirmed that this was driven by a significant association between MDS and Negative Affect at 10‐months; *r* (151) = 0.265, *p* < 0.001, [*CI* = 0.132, 0.395], but not at 16‐months; *r* (121) = −0.126, *p* = 0.166, [*CI* = −0.294, 0.039]. This association accounts for the significant main effect of assessment point found in this model (see Table 6), as results of a univariate *F*‐test of assessment point indicated that there was no significant difference between ratings of Negative Affect at 10‐ and 16‐ months; *F* (1, 140.401) = 0.507, *p* = 0.477 (as also indicated in Model 1).When ratings of Effortful Control were entered as the dependent variable, there were no significant main effects or interactions involving MDS, although there was still a significant main effect of assessment point (as also found in Model 1).

#### 3.4.3 Model 3: Influence of pandemic exposure on effortful control change from 10‐ to 16‐months

Since we observed a significant main effect of assessment point for ratings of Efforful Control in Model 2, we investigated whether the significant effect was associated with the duration of pandemic exposure. We decided to only run this model for the Effortful Control variable because, while we found a main effect of assessment point for ratings of Negative Affect, this was explained by the significant interaction with MDS.

Therefore, a further linear mixed model was conducted with length of pandemic exposure (days) as a fixed covariate and Effortful Control ratings as the dependent variable (Model 3, see Table 7). There was no significant main effect of pandemic exposure and no significant main effect of sub‐sample. There was also no significant assessment point × sub‐sample interaction, nor a significant assessment point × pandemic exposure interaction. However the main effect of assessment point remained significant.

**TABLE 7:** Model 3 (effortful control; length of pandemic exposure as fixed covariate): Estimated marginal means and type III fixed effects

Assessment point	***F* (1, 120.009) = 11.050, *p* = 0.001,** ${\mathrm{\eta p}}^{2}$ **= 0.084**
Sub‐sample	*F* (1, 128.467) = 0.075, *p* = 0.785, ${\mathrm{\eta p}}^{2}$ < 0.001
Assessment point × Sub‐sample	*F* (1, 120.009) = 0.181, *p* = 0.671, ${\mathrm{\eta p}}^{2}$ = 0.002
Pandemic exposure	*F* (1, 127.351) = 1.549, *p* = 0.216, ${\mathrm{\eta p}}^{2}$ = 0.012
Assessment point × Pandemic exposure	*F* (1, 118.640) = 0.069, *p* = 0.794, ${\mathrm{\eta p}}^{2}$ < 0.001
Assessment point	
10‐months	4.77 (0.062)
16‐months	5.08 (0.056)
Mean difference	0.304 (0.056)
Sub‐sample	
Pre‐pandemic	4.95 (0.092)
Pre‐ to during pandemic	4.90 (0.118)
Mean difference	0.050 (0.184)

*Note*: Mean (standard error) are reported. The length of pandemic exposure (days) covariate is entered into the model as 41.2283. Note that maximum likelihood estimation was used to account for missing data in this model, so all participants who contributed at least some data at either assessment point were included in these analyses.

Bold text indicates significant effects (*p* < .05).

## 4 INTERIM DISCUSSION: STUDY 1

Study 1 sought to investigate how ratings of MDS and infant temperament changed from 10‐ to 16‐months of age, and to reproduce previous findings of within‐age and longitudinal associations between MDS and temperament. Since the onset of the COVID‐19 pandemic fell during the 10‐ and 16‐month data collection period, we also aimed to assess the impact of the COVID‐19 pandemic on ratings of MDS, infant temperament, and their associations.

Before the pandemic, the link between maternal mood and infant expression of negative emotions and behaviours (i.e, Negative Affect) has been frequently demonstrated (Murray et al., 1996; Rigato, Stets, et al., 2020; Shapiro et al., 2018). Replicating this, data collected before the pandemic indicated that mothers with more depressive symptoms had 10‐month‐old infants who displayed higher levels of Negative Affect. Interestingly, by 16‐months this association had disappeared in both the pre‐ and during pandemic groups. Rigato, Stets, et al. (2020) proposed that the influence of MDS on infant Negative Affect would reduce from late infancy into toddlerhood due to the child's expanding social environment as they shift from maternal care to external childcare settings. Therefore, this appears to be a developmentally typical change in the association between MDS and Negative Affect from 10‐ to 16‐months that is not due to the pandemic. As Study 2 includes a larger sample of mothers (*N* = 220) with children that span a wider age range (6–48 months), we can further probe, with greater statistical power, the potential change in the association between MDS and Negative Affect across the early childhood period, albeit in the context of the pandemic.

While there was a noticeable lack of longitudinal associations between MDS and temperament at 10‐ and 16‐months, there was no evidence to suggest that this was a result of the pandemic. Similar research by Rigato, Stets, et al. (2020) before the pandemic identified moderate (*r* = 0.480) longitudinal associations between MDS and Negative Affect across a 6‐month period in infancy (but with younger infants from 4 to 9 months, which was the most similar age group to our study that also measures a 6‐month period in infancy). A post‐hoc power analysis (G*Power; Faul et al., 2007) with our smallest sample size for correlations (mean *N* = 63, pre‐pandemic group) suggested that we were well‐powered to detect correlational effects of this size (*r* = 0.480, 98% power). This suggests that, if longitudinal associations between MDS at 10‐months and temperament at 16‐months exist in this sample, they were too small to be detected. See Supporting Information 12 for full results of post‐hoc power calculations.

Results from the linear mixed models suggested that the pandemic had no effect on ratings of MDS and temperament, and that there was no change across assessment points in ratings of MDS or Negative Affect. A significant interaction between MDS and Negative Affect was found; however, this was due to the significant association between MDS and Negative Affect at 10‐months (before the pandemic), but not at 16‐months. While we observed an increase in ratings of Effortful Control from 10‐ to 16‐months, this was not related to pandemic exposure and would typically be expected at this point in development (Gartstein & Hancock, 2019; Gartstein & Rothbart, 2003; Putnam et al., 2006).

A post‐hoc power analysis (G*Power; Faul et al., 2007) revealed that our sample was well‐powered to detect medium (Cohen's *f* = 0.25, 89% power) effects when examining fixed effects. Therefore, while it is possible that ratings of MDS or Negative Affect did change over the pandemic time course, the effects would likely be weak and, as such, could not be detected due to limited statistical power for detecting small effects. Further research with a larger sample (thus greater statistical power) could support our understanding of whether more subtle pandemic effects influence MDS and child temperament over time. However, a clear implication of the current study is that there is no evidence for a *substantial* impact of the pandemic on these maternal and child variables, as may have been expected based on the amount of change that the pandemic caused in many people's lives.

In Study 2, we were able to assess whether changes in MDS and maternal stress *specifically* related to the pandemic had an impact the development of temperament across infancy and early childhood.

## 5 MATERIALS AND METHODS: STUDY 2

### 5.1 Participants

Participants (*N* = 286, 153 male children) were drawn from the sample of mothers of infants and young children (6–48 months) who were already involved in the Oxford Early Executive Functions (Oxford Early EF) study (described in Study 1 above) (*N* = 178, 98 males), or who had previously been involved in piloting for the Oxford Early EF study (*N* = 108, 55 male). As per the longitudinal study inclusion criteria, infants must be born at full‐term (at least 36 weeks) and/or with a minimum birth weight of 2500 g/5.5 pounds (*N* = 1 female excluded). Infants who experienced birth complications leading to health‐related concerns were excluded from all analyses (*N* = 4 males). Five mothers (male children) withdrew from the study before the second data collection window in November 2020.

A total of 220 participants (109 male children) contributed at least some questionnaire data to a minimum of one assessment point (Time 1: First national lockdown in April 2020 or Time 2: Second national lockdown in November 2020), constituting our study sample for Study 2. It is important to highlight that of the 220 participants, 154 (80 males) were involved in the Oxford Early Executive Functions (OEEF) study and, as such, also contributed data to Study 1 when their child was 10‐ and 16‐months of age. The remaining 66 participants were not part of the Oxford Early EF study and, as such, only contributed data to Study 2.

Participants in the reported sample were primarily from families of high to middle socioeconomic status, as indicated by the index of multiple deprivation decile (*M* = 7.6, SD = 2.12), annual household income (*M* = £76,014, SD = £35,477), and the mother's years in education (*M* = 18.26 years, SD = 3.28 years). The ethnicity of this sample was predominantly White British (64%). Complete demographic information for the reported sample is provided in Supporting Information 8.1.

### 5.2 Missing data

Full responses at both assessment points were received from 164 participants (80 male children). See Table 8 below for questionnaire response rates (see also Supporting Information 2.1 for further information). Comprehensive information abhout missing data in this study can be found in Supporting Information 2.2. According to the results of Little's missing completely at random test (Little, 1988) conducted in SPSS, the data missing in this study were missing completely at random; *χ* ^2^ (38) = 53.339, *p* = 0.050. See Supporting Information 2.2 for comprehensive information about missing data in this study including a missing data pattern analysis, and see also the Section 3.4 for how missing data were handled in our analyses.

**TABLE 8:** Questionnaire response rate by assessment point

	Time 1: April 2020	Time 2: November 2020
*N* of questionnaires sent	286	281
*N* of complete responses received	203	175
Complete response rate	72.4%	62.3%
*N* of partial responses received	14	0
Total *N* of responses received	217	175
Total response rate (partial or complete)	75.9%	62.3%

*Note*: Partial Responses refer to the receipt of questionnaire packs in which some, but not all questionnaires had been completed. Further information regarding the reasons why questionnaires were not sent to some participants at each assessment point are detailed in Supporting Information 2.1.

### 5.3 Materials

#### 5.3.1 Maternal depressive symptoms

The Beck Depression Inventory, second edition (BDI‐II; Beck et al., 1996) was completed by mothers as a measure of their depressive symptoms. Further details of this questionnaire, including measures of internal consistency from previous publications, are provided in Study 1. For this study (Study 2), the internal consistency (Cronbach's alpha) of the BDI‐II was excellent at Time 1 (April 2020) and Time 2 (November 2020), respectively; *α* = 0.92 and *α* = 0.93. See Supporting Information 4.1 for further details.

#### 5.3.2 Infant and child temperament

Mothers of infants aged 6–18 months completed the IBQ‐R‐VSF (Putnam et al., 2014) whereas mothers of children over 18 months completed the 36‐item Early Childhood Behaviour Questionnaire, Very Short Form (ECBQ‐VSF; Putnam et al., 2006). In both questionnaires, items relate to the child's behaviour in the last week. Scoring is the same as previously described for the IBQ‐R‐VSF in Study 1. Surgency and Negative Affect (also described in Study 1) are measured with the ECBQ‐VSF; however, Orienting/Regulatory Capacity is replaced with a measure of Effortful Control, which captures the ability to exert attentional control over behaviour and emotions. For simplicity throughout the article, we use the term “Effortful Control” when referring to the Orienting/Regulatory Capacity scale in the IBQ‐R‐VSF as these scales measure similar constructs.

In this study, the internal consistency of the three ECBQ‐VSF scales was acceptable and ranged from *α* =0.65 to *α* = 0.72, whereas the internal consistency (Cronbach's alpha) of the three IBQ‐R‐VSF scales ranged from 0.46 to 0.73 across both assessment points (Supporting Information 4.1). Since the Cronbach's alpha for the IBQ‐R‐VSF Surgency scale was too low to be considered reliable (*α* = 0.46), we do not include this scale in our analyses. However, results of analyses of the Surgency scale from the IBQ‐R‐VSF in Study 2 are reported in Supporting Information 10.

#### 5.3.3 A note on temperament questionnaire comparisons

As a result of the longitudinal nature of this study (see Table 1 and Figure 1) and the varying ages of the children at each time point (see Table 11 below), different temperament questionnaires were completed by some participants across the two pandemic assessment points (Time 1: April 2020, and Time 2: November 2020), with some completing the same questionnaire at both assessment points and others completing the IBQ‐R‐VSF in April (Time 1) and the ECBQ‐VSF in November (Time 2) due to the age of their child. Although the questionnaires measure similar temperament constructs, there are no overlapping items. While this enables questions to be asked about behaviours and characteristics that are developmentally appropriate to the child's age in infancy or toddlerhood, it also means that it is not possible to directly compare scores for the temperament constructs generated using different questionnaires (see Section 3.4 below for further information).

#### 5.3.4 COVID‐19 impact

The novel “Impact of COVID‐19 on Families with Young Children” questionnaire was used in the current study to assess the impact of the COVID‐19 pandemic on families with young children. This was sent to participants in April (Time 1) and November (Time 2) of 2020 in an online questionnaire pack which also included the MDS and temperament questionnaires. The COVID‐19 Impact questionnaire measures the effects of the pandemic on the family's physical and mental health, finances and employment, relationships, and the child's socioemotional and behavioural functioning. The full dataset from this questionnaire is available on OSF under a CC‐By Attribution 4.0 International licence [https://osf.io/zg97d/].

Ten items that capture the level of stress that the mother is under as a result of the COVID‐19 pandemic were compiled into a “COVID‐19 Stress Scale” (see Table 9). Questions asked about a range of negative feelings relating to the pandemic such as stress, worry, anxiety and depression. All questions were scored on a 5‐point scale, with answers ranging from 0 (e.g., “I am not feeling” or “I am not affected”) to 4 (e.g., “Extremely” or “Constantly”). A COVID‐19 stress score was created by summing the answers to each of the ten questions; the higher the score, the more stress the participant experienced. The internal consistency of this scale was good for the Time 1: April (*α* = 0.846) and Time 2: November datasets (*α* = 0.873). See Supporting Information 9 for further information on the COVID‐19 Stress Scale, including descriptive statistics and frequency histogram.

**TABLE 9:** Items in the COVID‐19 stress scale

Question	Rating
How worried are you feeling about the COVID‐19 pandemic?	
How low/depressed are you feeling about the COVID‐19 pandemic?	
How stressed are you feeling about the COVID‐19 pandemic?	
Have your feelings of anxiety increased due to the COVID‐19 pandemic?	0—I am not feeling… A ittle Somewhat Very Extremely
How worried are you feeling about your own physical health in the current situation?	
How worried are you feeling about your own mental health in the current situation?	
How worried are you feeling about a loved one's (family or close friend) physical health in the current situation?	
How worried are you feeling about a loved one's (family or close friend) mental health in the current situation?	
How much do you think about the negative outcomes/impact of the COVID‐19 pandemic on people's lives?	0—I do not think about… Occasionally Some of the time Most of the time Constantly
How badly do you think that you will be affected by the global effects of the COVID‐19 pandemic (e.g., reduced capacity of health care systems and global financial issues)?	0—Not at all affected Mildly affected Somewhat affected Moderately affected Severely affected

### 5.4 Procedure

Links to online Qualtrics questionnaire packs were sent to mothers via email at the start of the first national lockdown in the UK in April 2020 (Time 1), and again during the second national lockdown in November 2020 (Time 2). Due to the gradual recruitment at specific *age points* into these studies, children's ages varied because the data collection waves happened at *set points in time* (see Figure 1 and Table 10).

**TABLE 10:** Information on COVID‐19 pandemic data collection waves

	Time 1: First national lockdown (April 2020)	Time 2: Second national lockdown (November 2020)
Child's age when questionnaire sent	6–41 months	13–48 months
Child's sex	149 male, 137 female	149 male, 132 female
Data collection window	9th April–29th May 2020	23rd November 2020–11th January 2021
Data collection: *N* of days	51 days	50 days
*N* questionnaires sent	286 (153 male children)	281 (149 male children)
Complete responses	217 (111 male children)	175 (85 male children)
Partial responses	14 (4 male children)	0
Total response rate	75.9% (of 286 sent)	62.3% (of 281 sent)

### 5.5 Analysis approach

All analyses were conducted in SPSS version 27 and a two‐tailed significance level of *p* < 0.05 was used. Pearson's correlational analyses were performed to investigate longitudinal stability of individual differences, and to examine associations between the variables of interest, both within‐assessment points and longitudinally. Confidence intervals (95%) were calculated using bootstrapping (1000 samples).

When correlational analyses were conducted, no correction, estimation or imputation was carried out. Instead, data were excluded from analyses via pairwise deletion if data for one of the variables in the correlation were missing for a specific participant. For linear mixed model analyses, full information maximum likelihood estimation was used to account for missing data.

We conducted fully factorial linear mixed models to investigate change in ratings of MDS and temperament across assessment points (Time 1: April 2020 to Time 2: November 2020) (Model 1). We also examined change in temperament ratings across assessment points with MDS entered as a fixed covariate (Model 2). Finally, identical models were run with COVID‐19 stress as the fixed covariate (Model 3). This analysis approach was the same as in Study 1. However unlike Study 1 where participants' age was fixed at each assessment point, participants' ages varied in Study 2 and therefore we also included age as a fixed covariate in the temperament models. Since age (days) in April (Time 1) and November (Time 2) were very highly correlated; *r* (162) = 0.999, *p* < 0.001, [*CI* = 0.999, 0.999], we only entered age (days) in April (Time 1) as a covariate in the models.

In order to draw meaningful conclusions about changes in temperament ratings and associations with MDS over time, our analyses focused on data that were collected from the same temperament questionnaire (i.e., no cross‐questionnaire comparisons were made). Data entered in the temperament models and correlations were from participants who completed the ECBQ‐VSF at both assessment points (*N* = 119). Although part of the sample completed the IBQ‐R‐VSF at both assessment points, the small sample size (*N* = 21) and resulting inadequate statistical power means that it is difficult to accurately interpret any results from this group.

## 6 RESULTS: STUDY 2

### 6.1 COVID‐19 impact questionnaire

**FIGURE 2:**
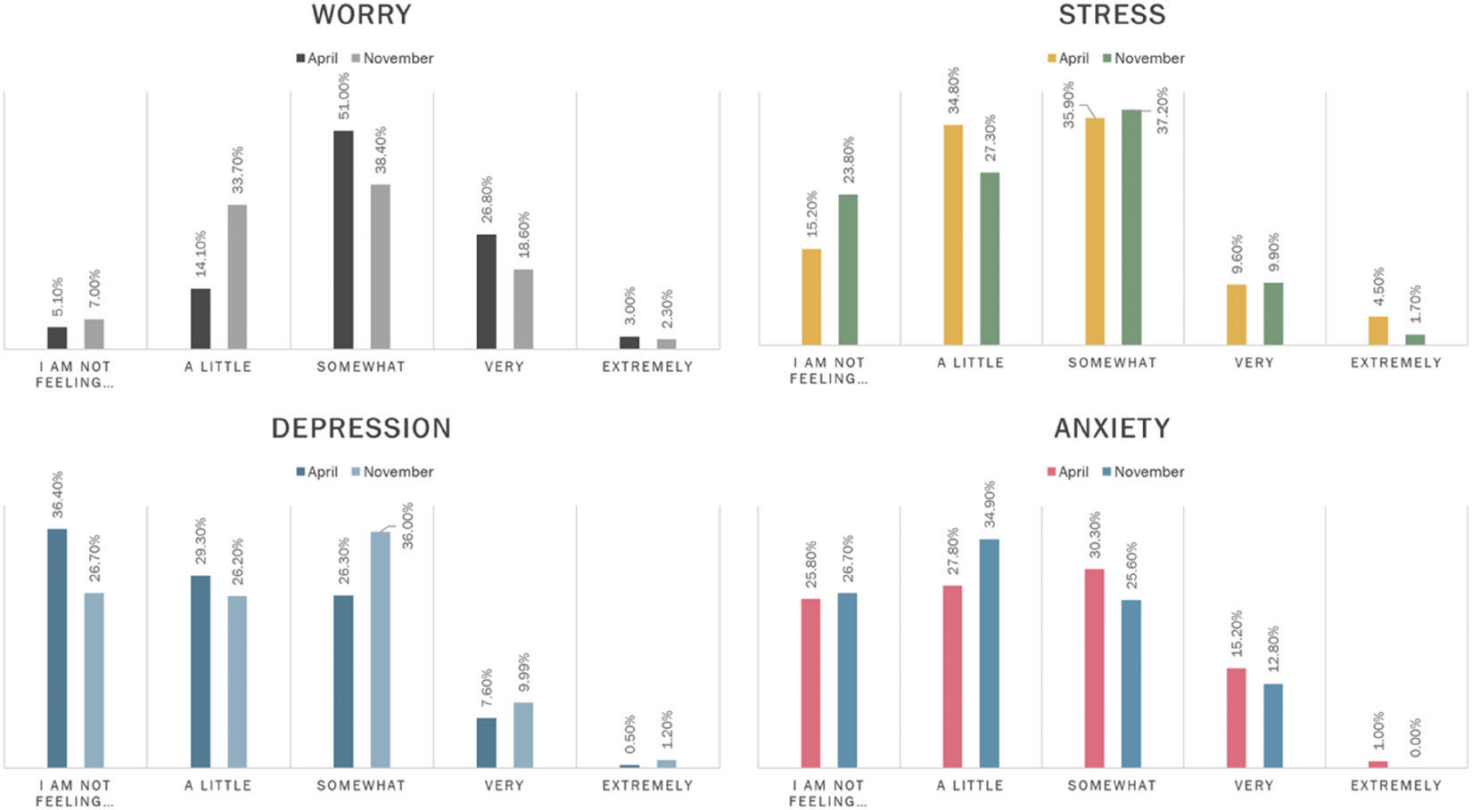
Maternal pandemic‐related worry, stress, depression and anxiety during the 2020 UK National Lockdowns. Each graph represents the score on a single item in the COVID‐19 Stress scale at both assessment points

During the first national lockdown in April 2020 (Time 1), the majority of mothers (63%–94%) reported feeling *some* level of worry, stress, anxiety and depression as a specific result of the COVID‐19 pandemic (see Figure 2). The overall pandemic‐specific COVID‐19 stress scale score (i.e., the combined score across all items in the scale) was significantly higher in April (Time 1: *M* = 14.85, *SD* = 6.29, *N* = 197) than in November (Time 2: *M* = 14.07, *SD* = 6.82, *N* = 173); Z = −2.026, *p* = 0.043, *d* = 0.160. However, mothers reporting at least some level of depression related to the pandemic (a single item in the questionnaire) had significantly increased by the second lockdown; *t* (158) = 2.435, *p* = 0.016, *d* = 0.193.

Pearson's correlational analyses were conducted to investigate longitudinal and within‐assessment point associations between MDS (as measured with the BDI‐II) and the COVID‐19 stress scale score. Results are reported in Table 11 and suggest that moderate concurrent and predictive associations exist between the two measures. A Wilcoxon signed rank test confirmed that there was no significant difference between mothers' ratings of their depressive symptoms (as measured with the BDI‐II) in the first national lockdown in April (*M* = 9.60, *SD* = 8.44, *N* = 207) and the second in November (*M* = 9.73, *SD* = 8.79, *N* = 173); *Z* = 0.559, *p* = 0.576, *d* = 0.044.

**TABLE 11:** Longitudinal and concurrent associations between MDS and COVID‐19 stress

	April COVID‐19 stress	April MDS	November COVID‐19 stress	November MDS
April COVID‐19 stress	–			
April MDS	0.505*** [0.373, 0.612] *161*	–		
November COVID‐19 Stress	0.612*** [0.515, 0.710] *161*	366*** [0.241, 0.495] *161*	–	
November MDS	0.377*** [0.233, 0.507] *161*	0.750*** [0.624, 0.845] *161*	0.446*** [0.316, 0.569] *161*	–

*Note*: MDS was measured using the BDI‐II. COVID‐19 Stress refers to scores on the COVID‐19 Stress scale (see Table 9 in Section 3.2). Cells show the correlation coefficient, 95% confidence intervals are reported in square brackets, and *N* in italics. Note that pairwise deletion was used such that only participants who contributed data to both variables in the correlation pair were included in analyses. All associations remained significant when controlling the false discovery rate.

*** *p* < 0.001.

### 6.2 MDS and temperament during the first and second national lockdowns in 2020

Descriptive statistics from the full sample (*N* = 220) are provided for ratings of temperament across all questionnaires (Table 12). However, we only analysed temperament data from participants who completed the ECBQ‐VSF at both assessment points (*N* = 119). See Sections 3.2 and 3.4 for information on our decision not to make cross‐questionnaire comparisons.

**TABLE 12:** Descriptive statistics for ratings of temperament by questionnaire

	*N*	IBQ‐R‐VSF	*N*	ECBQ‐VSF	*N*	IBQ‐R‐VSF (time 1) and ECBQ‐VSF (time 2)
Age of child (months)						
Time 1: April	21	8.95 (1.75)	119	25.75 (6.95)	71	14.31 (2.09)
Time 2: November	17	16.24 (1.72)	96	31.94 (6.19)	60	21.82 (2.03)
Surgency^a^						
Time 1: April	–	–	119	5.23 (0.57)	71	–
Time 2: November	–	–	96	5.32 (0.62)	60	5.23 (0.62)
Negative affect						
Time 1: April	21	3.99 (0.82)	119	2.72 (0.71)	71	4.03 (0.93)
Time 2: November	17	3.98 (0.70)	96	2.76 (0.63)	60	2.85 (0.62)
Effortful control						
Time 1: April	21	4.78 (0.61)	119	4.55 (0.68)	71	5.12 (0.62)
Time 2: November	17	4.89 (0.68)	96	4.80 (0.61)	60	4.48 (0.72)

*Note*: Data in table represent the mean (standard deviation). For the group that completed a different questionnaire at each assessment point, the temperament means are not comparable across assessment points due to the change in scale.

Abbreviations: ECBQ‐VSF, Early Childhood Behaviour Questionnaire—Very Short Form; IBQ‐R‐VSF, Infant Behaviour Questionnaire—Revised, Very Short Form.

^a^ Data pertaining to the Surgency scale of the IBQ‐R‐VSF are not reported here due to the poor internal consistency of this scale; see Supporting Information 10 for these results.

### 6.3 Longitudinal stability

Results of Pearson's correlation analyses indicated strong longitudinal stability of individual differences in MDS and child temperament dimensions across assessment points (Table 13). Recall that our analyses focused on data that were collected from the same temperament questionnaire (ECBQ‐VSF) such that no cross‐questionnaire comparisons were made. There was also significant longitudinal stability in ratings of COVID‐19 stress from April to November 2020 (Table 11). All correlations remained significant when controlling the false discovery rate.

**TABLE 13:** Longitudinal and concurrent associations between ratings of MDS and child temperament from time 1 (April 2020) to time 2 (November 2020)

	April MDS	April Surgency	April NA	April EC	Nov MDS	Nov Surgency	Nov NA	Nov EC
April: MDS	–							
April: Surgency	0.192 * [0.003, 0.362) *115*	*–*						
April: Negative Affect (NA)	0.101 [−0.073, 0.312] *115*		–					
April: Effortful Control (EC)	−0.190 * [−0.359, −0.015] *115*			–				
November MDS	0.768***, ^a^ [0.650, 0.844] *164*	0.245 **, ^a^ [0.039, 0.411] *89*	0.211 * [0.025, 0.430] *89*	−0.246 *, ^a^ [−0.444, −0.062] *89*	–			
November Surgency	0.071 [−0.094, 0.232] *147*	0.539 ***, ^a^ [0.372, 0.679] *89*			0.009 [−0.164, 0.170] *148*	*–*		
November NA	0.040 [−0.111, 0.222] *147*		0.633 ***, ^a^ [0.502, 0.742] *89*		0.137 [−0.025, 0.315] *148*		–	
November EC	−0.190 ** [−0.331, −0.047] *147*			0.606 ***, ^a^ [0.448, 0.735] *89*	−0.222 **, ^a^ [−0.374, −0.075] *148*			–

*Note*: Cells show the correlation coefficient, 95% confidence intervals in square brackets, and *N* in italics. Blank cells indicate correlations that were not run. Underlined coefficients are partial correlations controlling for age (days) in April 2020. Note that pairwise deletion was used such that only participants who contributed data to *both* variables in the correlation pair were included in analyses. Temperament data are from participants who completed the ECBQ‐VSF at both assessment points.

^a^ Remained significant following the procedure for controlling the false discovery rate.

* *p* < 0.05;

** *p* < 0.01;

*** *p* < 0.001.

### 6.4 Concurrent associations between MDS, COVID‐19 stress and child temperament

Within each assessment point (Time 1: April 2020, Time 2: November 2020), partial correlation analyses were conducted to investigate associations between MDS and child temperament when controlling for age (days) in April 2020; results are reported in Table 13. We did not find any significant associations between MDS and Negative Affect within either assessment point. A small, positive association was found between MDS and Surgency in April, however by November this association was no longer significant. There was a significant negative association between MDS and Effortful Control in both April and November when controlling for age, however only the association in November remained significant when controlling the false discovery rate.

Identical analyses were also conducted to investigate concurrent associations between maternal COVID‐19 stress and temperament ratings (controlling for age), however no significant correlations were found; results are reported in Supporting Information 11.

### 6.5 Longitudinal associations between MDS and child temperament

To investigate potential longitudinal associations between MDS and temperament, partial correlation analyses were conducted across assessment points, controlling for age (Table 13). Similar partial correlation analyses were run to investigate longitudinal associations between COVID‐19 stress and temperament, however there were no significant effects (Supporting Information 11).

A small, positive association was found between ratings of child Surgency in April and MDS in November, which remained significant when controlling the false discovery rate. However, this association was no longer significant when additionally controlling for MDS in April; *r* (84) = 0.105, *p* = 0.334, [*CI* = −0.110, 0.296]. A small, positive association was found between Negative Affect in April and MDS in November (although this did not retain significance when controlling the false discovery rate). When also controlling for MDS in April, the association remained significant; *r* (84) = 0.238, *p* = 0.027, [*CI* = 0.042, 0.434].

A small, negative correlation was found between ratings of the child's Effortful Control in April and MDS in November, and remained significant when controlling the false discovery rate. When additionally controlling for MDS in April; however, this association was no longer significant; *r* (84) = −0.187, *p* = 0.085, [*CI* = −0.398, 0.035]. Similarly, a weak negative correlation was found between MDS in April and Effortful Control in November. This correlation remained significant when controlling the false discovery rate. When also controlling for MDS in November, the correlation was no longer significant; *r* (143) = −0.037, *p* = 0.658, [*CI* = −0.210, 0.118].

### 6.6 Change in ratings of MDS and temperament from April to November 2020

#### 6.6.1 Model 1: Change in MDS and temperament ratings from April to November 2020

In Table 14 we report the estimated marginal means and results of Type III fixed effect tests for the models that included assessment point (Time 1: April 2020, Time 2: November 2020) as a fixed factor, participants as a random factor, and age in days in April 2020 as a fixed covariate (Model 1). There was no significant effects when MDS, Negative Affect or Effortful Control were entered into the model as the dependent variables. When Surgency was entered into the model, there was no significant main effect of assessment point or age; however, there was a significant assessment point × age interaction. Post‐hoc correlational analyses revealed a significant association between Surgency and age in April; *r* (112) = −0.216, *p* = 0.021, [*CI* = −0.385, −0.046], with higher Surgency reported for younger children in April, but not in November; *r* (144) = 0.091, *p* = 0.273, [*CI* = −0.084, 0.277].

**TABLE 14:** Model 1: Type III fixed effects and estimated marginal means

	MDS	Surgency	Negative affect	Effortful control
Assessment point	*F* (1, 178.255) = 0.820, *p* = 0.366, ${\mathrm{\eta p}}^{2}$ < 0.001	***F* (1, 94.548) = 7.101,** ***p* = 0.009,** ${\mathrm{\eta p}}^{2}$ **= 0.070**	*F* (1, 94.966) = 1.433, *p* = 0.234, ${\mathrm{\eta p}}^{2}$ = 0.015	*F* (1, 101.664) = 2.216, *p* = 0.140, ${\mathrm{\eta p}}^{2}$ = 0.021
Age	–	*F* (1, 121.446) = 0.065, *p* = 0.799, ${\mathrm{\eta p}}^{2}$ = 0.000	*F* (1, 119.007) = 2.442, *p* = 0.121, ${\mathrm{\eta p}}^{2}$ = 0.020	*F* (1, 125.630) = 0.084, *p* = 0.773, ${\mathrm{\eta p}}^{2}$ = 0.000
Assessment point × Age	–	***F* (1, 95.142) = 8.973, *p* = 0.003,** ${\mathrm{\eta p}}^{2}$ **= 0.086**	*F* (1, 96.050) = 0.853, *p* = 0.358, ${\mathrm{\eta p}}^{2}$ = 0.009	*F* (1, 102.780) = 0.129, *p* = 0.721, ${\mathrm{\eta p}}^{2}$ = 0.001
April estimated marginal mean	9.45 (0.571)	5.24 (0.052)	2.72 (0.063)	4.54 (0.062)
November estimated marginal mean	9.87 (0.611)	5.31 (0.063)	2.79 (0.066)	4.81 (0.062)
Mean difference	0.418 (0.461)	0.070 (0.058)	0.068 (0.057)	0.275 (0.059)

*Note*: Mean (standard error) are reported. Temperament data in table are only from the ECBQ‐VSF. Covariates entered into the temperament models: Age (days) = 786.86. Note that maximum likelihood estimation was used to account for missing data in this model, so all participants who contributed at least some data at either assessment point were included in these analyses.

Bold text indicates significant effects (*p* < .05).

#### 6.6.2 Model 2: Influence of MDS on temperament ratings from April to November 2020

Table 15 contains the estimated marginal means and results of the Type III fixed effects tests for the temperament models in which MDS was entered as a fixed covariate (Model 2) alongside age (in days) in April 2020. When Surgency was entered into the model as the dependent variable, there was no influence of MDS on change in ratings across assessment points; however, a significant main effect of assessment point and a significant assessment point × age interaction was found. Note that a significant assessment point × age interaction was also found in Model 1 and was driven by a significant association between Surgency and age in April, but not in November. There was no significant change in ratings of Negative Affect across assessment points, and no significant main effect or interaction involving the age or MDS covariates. When Effortful Control was entered into the model as the dependent variable, there were no significant main effects or interactions.

**TABLE 15:** Model 2: Type III fixed effects and estimated marginal means

	Surgency	Negative affect	Effortful control
Assessment point	***F* (1, 90.607) = 4.059, *p* = 0.047,** ${\mathrm{\eta p}}^{2}$ **= 0.043**	*F* (1, 92.438) = 0.602, *p* = 0.440, ${\mathrm{\eta p}}^{2}$ = 0.006	*F* (1, 98.031) = 1.773, *p* = 0.186, ${\mathrm{\eta p}}^{2}$ = 0.018
MDS	*F* (1, 158.269) = 0.243, *p* = 0.623, ${\mathrm{\eta p}}^{2}$ = 0.002	*F* (1, 183.304) = 1.924, *p* = 0.167, ${\mathrm{\eta p}}^{2}$ = 0.010	*F* (1, 174.841) = 0.156, *p* = 0.694, ${\mathrm{\eta p}}^{2}$ < 0.001
Age	*F* (1, 132.875) = 0.030, *p* = 0.863, ${\mathrm{\eta p}}^{2}$ < 0.001	*F* (1, 148.139) = 0.137, *p* = 0.712, ${\mathrm{\eta p}}^{2}$ = 0.001	*F* (1, 145.369) = 0.003, *p* = 0.954, ${\mathrm{\eta p}}^{2}$ < 0.001
Assessment point × MDS	*F* (1, 90.649) = 0.229, *p* = 0.633, ${\mathrm{\eta p}}^{2}$ = 0.003	*F* (1, 91.559) = 0.016, *p* = 0.900, ${\mathrm{\eta p}}^{2}$ < 0.001	*F* (1, 96.607) = 0.324, *p* = 0.571, ${\mathrm{\eta p}}^{2}$ = 0.003
Assessment point × Age	***F(*1, 90.077) = 6.663, *p* = 0.011,** ${\mathrm{\eta p}}^{2}$ **= 0.069**	*F* (1, 92.140) = 0.258, *p* = 0.613, ${\mathrm{\eta p}}^{2}$ = 003	*F* (1, 97.733) = 0.291, *p* = 0.591, ${\mathrm{\eta p}}^{2}$ = 0.003
MDS × Age	*F* (1, 150.232) = 0.118, *p* = 0.732, ${\mathrm{\eta p}}^{2}$ < 0.001	*F* (1, 175.105) = 2.720, *p* = 0.101, ${\mathrm{\eta p}}^{2}$ = 0.015	*F* (1, 166.764) = 0.041, *p* = 0.839, ${\mathrm{\eta p}}^{2}$ < 0.001
Assessment point × MDS × Age	*F* (1, 89.486) = 0.808, *p* = 0.371, ${\mathrm{\eta p}}^{2}$ = 0.009	*F* (1, 90.795) = 0.002, *p* = 0.967, ${\mathrm{\eta p}}^{2}$ < 0.001	*F* (1, 95.722) = 0.229, *p* = 0.634, ${\mathrm{\eta p}}^{2}$ < 0.001
April estimated marginal mean	5.23 (0.051)	2.71 (0.062)	4.53 (0.062)
November estimated marginal mean	5.30 (0.064)	2.79 (0.065)	4.81 (0.060)
Mean difference	0.071 (0.059)	0.079 (0.057)	0.278 (0.059)

*Note*: Mean (standard error) are reported for each of the estimated marginal means. Data in table are only from the ECBQ‐VSF. Covariates entered into the model: MDS = 9.71, Age (days) = 783.21. Note that maximum likelihood estimation was used to account for missing data in this model, so all participants who contributed at least some data at either assessment point were included in these analyses.

Bold text indicates significant effects (*p* < .05).

#### 6.6.3 Model 3: Influence of maternal COVID‐19 stress on change in temperament ratings from April to November 2020

No significant effects or interactions involving COVID‐19 stress were found in models with the temperament dimensions as the dependent variable (Table 16). However, a significant mean increase for ratings of Surgency and a significant assessment point × age interaction was still found (as in Models 1 and 2). When Effortful Control was entered into the model as a dependent variable, there were no significant main effects or interactions.

**TABLE 16:** Model 3: Type III fixed effects and estimated marginal means

	Surgency	Negative affect	Effortful control
Assessment point	***F* (1, 104.054) = 5.281, *p* = 0.024,** ${\mathrm{\eta p}}^{2}$ **= 0.048**	*F* (1, 101.214) = 1.239, *p* = 0.258, ${\mathrm{\eta p}}^{2}$ = 0.012	*F* (1, 108.443) = 0.341, *p* = 0.561, ${\mathrm{\eta p}}^{2}$ = 0.003
COVID‐19 stress	*F* (1, 181.562) = 0.150, *p* = 0.699, ${\mathrm{\eta p}}^{2}$ < 0.001	*F* (1, 196.306) = 0.415, *p* = 0.520, ${\mathrm{\eta p}}^{2}$ = 0.002	*F* (1, 195.738) = 2.536, *p* = 0.113, ${\mathrm{\eta p}}^{2}$ = 0.013
Age	*F* (1, 181.919) = 1.094, *p* = 0.297, ${\mathrm{\eta p}}^{2}$ = 0.006	*F* (1, 196.085) = 1.112, *p* = 0.293, ${\mathrm{\eta p}}^{2}$ = 0.006	*F* (1, 194.173) = 1.509, *p* = 0.221, ${\mathrm{\eta p}}^{2}$ = 0.008
Assessment point × COVID‐19 stress	*F* (1, 100.838) = 1.542, *p* = 0.217, ${\mathrm{\eta p}}^{2}$ = 0.015	*F* (1, 99.235) = 2.944, *p* = 0.089, ${\mathrm{\eta p}}^{2}$ = 0.029	*F* (1, 105.148) = 1.231, *p* = 0.270, ${\mathrm{\eta p}}^{2}$ = 0.012
Assessment point × Age	***F* (1, 106.781) = 5.345, *p* = 0.023,** ${\mathrm{\eta p}}^{2}$ **= 0.048**	*F* (1, 103.483) = 1.544, *p* = 0.217, ${\mathrm{\eta p}}^{2}$ = 0.015	*F* (1, 110.002) = 1.046, *p* = 0.309, ${\mathrm{\eta p}}^{2}$ = 0.009
COVID‐19 stress × Age	*F* (1, 182.399) = 0.621, *p* = 0.432, ${\mathrm{\eta p}}^{2}$ = 0.003	*F* (1, 195.521) = 0.334, *p* = 0.564, ${\mathrm{\eta p}}^{2}$ = 0.002	*F* (1, 195.601) = 1.779, *p* = 0.184, ${\mathrm{\eta p}}^{2}$ = 0.009
Assessment point × COVID‐19 stress × Age	*F* (1, 102.461) = 1.260, *p* = 0.264, ${\mathrm{\eta p}}^{2}$ = 0.012	*F* (1, 100.915) = 2.883, *p* = 0.093, ${\mathrm{\eta p}}^{2}$ = 0.028	*F* (1, 106.860) = 1.293, *p* = 0.258, ${\mathrm{\eta p}}^{2}$ = 0.012
April estimated marginal mean	5.21 (0.052)	2.69 (0.065)	4.53 (0.062)
November estimated marginal mean	5.32 (0.063)	2.78 (0.064)	4.79 (0.062)
Mean difference	0.107 (0.062)	0.084 (0.060)	0.264 (0.060)

*Note*: Mean (standard error) are reported for each of the estimated marginal means. Data in table are only from the ECBQ‐VSF. Covariates entered into the model: COVID‐19 stress = 14.53, Age (days) = 775.24. Note that maximum likelihood estimation was used to account for missing data in this model, so all participants who contributed at least some data at either assessment point were included in these analyses.

Bold text indicates significant effects (*p* < .05).

## 7 INTERIM DISCUSSION: STUDY 2

With this study, we aimed to investigate the impact of the COVID‐19 pandemic on MDS, child temperament and aspects of maternal mental health related specifically to the pandemic. We observed strong evidence of longitudinal stability in individual differences in MDS and child temperament over time and, against our prediction, found no significant increase in MDS or Negative Affect across our pandemic assessment points (April 2020 and November 2020). Overall, we found no evidence of pandemic‐specific impacts on MDS and early childhood temperament.

However, we did find evidence of associations between MDS and aspects of child temperament across the pandemic assessment points. This is in contrast to Study 1, where no longitudinal associations were found between MDS and infant temperament, regardless of pandemic exposure. The larger sample size available for correlational analyses in Study 2 meant that we were better powered (92.33%) to detect moderate effects (*r* = 0.30); see Supporting Information 12 for full results of post‐hoc power calculations for this study.

Interestingly, we observed child‐driven longitudinal associations between Surgency, Negative Affect and Effortful Control in April and MDS in November. Children with higher levels of Negative Affect and Surgency, and lower levels of Effortful Control at the first assessment point had mothers with more depressive symptoms later in the pandemic. This hints at the predictive influence of child temperament on later MDS and provides some evidence that the link between maternal mood and temperament in early childhood may be child‐driven, not maternally‐driven as in infancy (Rigato, Stets, et al., 2020; Shapiro et al., 2018). Since these longitudinal associations between MDS and temperament were of a small effect size (minimum *r =* 0.190, maximum *r =* 0.246), replication of these results in a larger sample is needed to confirm the reliability of this preliminary evidence.

We also found that the COVID‐19 stress index decreased from April 2020 (first national lockdown) to November 2020 (second national lockdown), suggesting that mothers' level of pandemic‐related stress reduced as the pandemic continued. This is in contrast to mothers' ratings of their *pandemic‐related* feelings of depression, which significantly increased from April to November. While these results may suggest that there was an increase in mothers subjective reports of depressive symptoms associated specifically with the pandemic over time, this result should be interpreted with caution as the effect was not detected when assessing depressive symptoms using the BDI‐II; a gold‐standard measure of depression. It is worth considering that the pandemic‐related depression was measured from a single questionnaire item, whereas the BDI‐II is a full 21‐item scale, with high internal consistency, measuring more global depressive symptoms. Overall, our results indicated that maternal pandemic‐related stress did not influence the development of child temperament.

## 8 GENERAL DISCUSSION

The current study investigated the development of temperament and associations with MDS across infancy and early childhood, before and during the COVID‐19 pandemic. In Study 1 we found increases in Effortful Control ratings from 10‐ to 16‐months of age, and an association between MDS and Negative Affect at 10‐months; however, no pandemic‐specific effects were found. In Study 2, we found some evidence of child‐driven associations between child temperament early in the pandemic and MDS 7 months later, as well as bi‐directional associations between MDS and Effortful Control in early childhood. Consistent with existing research, results from both studies demonstrated that, even during the pandemic, we find the expected longitudinal stability in mothers' ratings of their depressive symptoms (Rigato, Stets, et al., 2020; Takács et al., 2019) and of their child's temperament (Carranza et al., 2013; Putnam et al., 2006, 2008; Rothbart et al., 2000).

Although no pandemic‐specific impact was found, our results contribute to knowledge on early temperament development by demonstrating a shift in association between MDS and child temperament across the transition from infancy to early childhood. Considering evidence from previous literature (e.g., Rigato, Stets, et al., 2020) along with data from both of the current studies, we propose that while MDS are positively associated with Negative Affect in infancy, this association weakens into toddlerhood where a new negative association between MDS and Effortful Control appears. In Study 1, mothers with more DS had 10‐month‐old infants who displayed higher levels of Negative Affect, yet by 16‐months, this association had disappeared. In Study 2 (where the majority of children in the sample were over 18 months), we found no significant within‐age association between MDS and Negative Affect, but instead observed that mothers with more depressive symptoms concurrently had children with lower ratings of Effortful Control at both assessment points.

Also in Study 2, we captured the possible emergence of bi‐directional associations between MDS and Effortful Control across the early childhood period. We found that mothers with more depressive symptoms early in the pandemic had children (regardless of age) displaying lower Effortful Control 7 months on, and children displaying lower Effortful Control early in the pandemic having mothers with more depressive symptoms later in the pandemic. The longitudinal association between MDS and Effortful Control was no longer significant when also controlling for MDS in November, thus hinting at a more global association between MDS and Effortful Control across the two assessment points. Since these associations were unaffected by pandemic‐related stress, it is possible that we have uncovered a change in the ways in which MDS interact with temperament dimensions from infancy to early childhood. Indeed, previous studies have highlighted associations between MDS and the development of Effortful Control in older children (Choe et al., 2014; Pesonen et al., 2006). Nonetheless, further research is needed that investigates how associations between MDS, child Negative Affect and Effortful Control change from the end of the first year and into early childhood, in order to establish whether our results would replicate outside the pandemic context.

Furthermore, while evidence from Study 1 suggests that there is a spurt in the development of Effortful Control across the transition from late infancy to toddlerhood (i.e., from 10‐ to 16‐months), the same kind of growth does not appear to be present after this age. In Study 2, when the children in the sub‐sample were older than 18‐months, we did not find evidence of an increase in Effortful Control rating across the 7‐month window between assessment points. This could be explained by the large age spread of participants (18–48 months) and so the individual differences in Effortful Control may have masked any overall increase across assessment points. Similarly, the change in temperament measure from Study 1 (IBQ‐R‐VSF) to Study 2 (ECBQ‐VSF) may have had an influence on whether a change in Effortful Control across assessment points was observed.

Importantly, we did not observe a significant increase in MDS in the proportion of mothers who experienced the pandemic by the time their infant was 16‐months old, or in mothers of young children across the first 7 months of the COVID‐19 pandemic. These findings were against our expectations and contradict an emerging literature which suggests increased MDS during the COVID‐19 pandemic (Cameron et al., 2020; Davenport et al., 2020; Lebel et al., 2020; Racine et al., 2021; Wu et al., 2020). Although there is currently limited research that specifically investigates the impact of the COVID‐19 pandemic on early childhood temperament, our finding of a general lack of pandemic‐related impacts is largely in line with research investigating the effects of natural disaster‐related stress on infant temperament. Like us, Tees et al. (2010) and Buthmann et al. (2019) found no evidence of associations between mothers' subjective disaster‐related stress and infant temperament. Additionally, in a recent meta‐analysis of studies investigating MDS during the COVID‐19 pandemic, Hessami et al. (2020) failed to find a significant increase in MDS during the pandemic in a pooled population of over 7000 expectant or new mothers, in contrast with a robust effect on anxiety. Since some pandemic research has suggested that mothers with prior psychopathology have a significantly greater risk of experiencing depressive symptoms during the pandemic (Cameron et al., 2020; Racine et al., 2021), it is possible that in a sample with low levels of MDS prior to the pandemic, such increases would not be observed.

It is key to note that potential pandemic effects on both child temperament and maternal well‐being might be crucially moderated by the age of children sampled by individual studies. Many during‐pandemic studies focus on mothers during pregnancy or the early post‐partum period (e.g., Filippetti et al., 2022) and may be capturing an elevation of pregnancy‐related stressors or depressive symptoms as a result of negative pandemic‐related changes, such as difficulty in accessing healthcare and social support. Other studies surveyed mothers of school‐aged children, who likely faced an entirely different set of challenges during the pandemic, such as the pressures of school closures, home‐schooling and financial difficulties from reducing their own work hours (e.g., Racine et al., 2021). Indeed, in preschool children there may be additional effects on temperament that come from changes associated with preschool closures that could contribute to the disparity in observing pandemic‐related effects in early childhood, compared with school‐aged children—see Davies et al. (2021) for a relevant discussion on the impact of pandemic‐related closures of early childhood education and care centres on young children's development.

Gathering knowledge about the differential impact of the COVID‐19 pandemic on diverse groups within the population is fundamental to identifying where support may be needed in order to alleviate any negative pandemic consequences. Our sample bridged the gap in research between the infancy and school age‐period (0–4 years) and, as such, may have captured a group in which there is a reduced or no pandemic impact on both child temperament and maternal difficulties. However, since our sample was sufficiently powered to detect only medium and large effects (see Supporting Information 12 for post‐hoc power calculations), it is possible that more subtle effects of the pandemic may not have been detected and so future research with larger samples is needed to confirm these results. Overall, our results provide encouraging preliminary evidence that the well‐being of mothers with young children and temperament development in infancy and early childhood are largely unaffected by the COVID‐19 pandemic, at least in the early stage of the pandemic and in this specific sample demographic.

### 8.1 Strengths and limitations of the current study

A substantial strength of the current research is the availability of the 10‐ and 16‐month infant data in Study 1 which was collected before the pandemic. This is unlike many during‐pandemic studies that rely on participants to provide retrospective reports on psychological variables before the pandemic (Davenport et al., 2020), or that compare data from two different participant populations cross‐sectionally (Cameron et al., 2020). Our pre‐pandemic data therefore allow us to draw conclusions about the actual impact of the pandemic on MDS and infant temperament in Study 1. While we recognize the potential limitations that parent‐reported data may incur, we would expect that rater‐bias would affect both the pre‐ and during‐pandemic data to the same extent. Additionally, by using online questionnaires, we have been able to safely collect important data on the development of early childhood temperament during a global pandemic which otherwise we would simply not have. As a result, we were able to investigate, during the early stages of the pandemic, the potential short‐term impacts of the pandemic on MDS and child temperament. To capture the possible longer‐term impacts, further data collection is ongoing and will examine the continued effects of the pandemic on MDS and early childhood temperament.

In Study 2, we were limited by the transition from the IBQ‐R‐VSF (infants under 18 months) to the ECBQ‐VSF (children over 18 months), which meant that although we had pre‐pandemic temperament data from most participants, for the majority, this was collected using a different measure that cannot be directly compared with the ECBQ‐VSF. While this change of scale was necessary to keep up with children's development, this meant that we were unable to conclude whether the observed longitudinal effects in Study 2 were pandemic‐specific or a result of typical development at this point in early childhood. Future research that assesses the continuity of temperament using a consistent questionnaire or measurement tool across development would pave the way for longitudinal studies that examine temperament trajectories across infancy and early childhood, without having to limit comparisons to age ranges where a specific measure is age‐appropriate (see, for example, Hendry & Holmboe, 2020).

Unfortunately, despite a consistent scheme of communication with parents to encourage questionnaire completion, the attrition that is common in most longitudinal studies was somewhat worse during the pandemic period, and we cannot rule out that the mothers who did not participate during the pandemic were the mothers who were experiencing higher DS and pandemic‐related stress. It is not possible to know whether the inclusion of these mothers might have led to the uncovering of important associations that we did not find in the current sample of mothers.

It is also important to consider that participants in this study were generally from well‐educated, primarily White British backgrounds, and were living in an affluent university city in the UK, and as such may be benefitting from the protective influence of a number of resilience factors relating to familial and societal context (Cadamuro et al., 2021; Gordon‐Hollingsworth et al., 2018; Peek, 2008). Significant demographic and psycho‐social risk factors could not be examined in depth in this study due to the sample's restricted variation on these variables. Emerging evidence indicates that parents who were unemployed or from low‐income households reported higher anxiety and depression levels during the pandemic than those who were employed or had a higher income (McElroy et al., 2020; Shum et al., 2020). Additionally, research suggests that young children living in poorer socio‐economic circumstances may have been more affected by the pandemic unless they were buffered by available early childhood care support structures, compared with children living in higher income brackets (Davies et al., 2021). Overall, this evidence indicates that multiple factors beyond immediate family factors contribute to the impact on individual children. We therefore encourage additional studies during and beyond the pandemic that can help to establish whether the resilience of temperament development found in this study also applies in more disadvantaged and at‐risk populations of mothers and children, both throughout the UK and across the globe.

## 9 CONCLUSION

This study investigated levels of MDS and the development of temperament in infants and young children in the United Kingdom before and during the COVID‐19 pandemic in 2020. Contrary to our expectations, we found no substantial impact of the pandemic on the typical development of temperament across infancy and early childhood. We also found no evidence of increases in MDS during the pandemic. In a time where we are faced with many negative consequences of the COVID‐19 pandemic, this longitudinal study demonstrates the robustness of normative temperament development in infancy and early childhood.

## AUTHOR CONTRIBUTIONS

**Abigail Fiske:** Conceptualization; data curation; formal analysis; investigation; methodology; project administration; validation; visualization; writing – original draft; writing – review and editing. **Gaia Scerif:** Formal analysis; supervision; writing – review and editing. **Karla Holmboe:** Conceptualization; data curation; formal analysis; funding acquisition; methodology; project administration; supervision; writing – review and editing.

## FUNDING INFORMATION

This research was funded by the UK Medical Research Council (MR/N008626/1; PI: K. Holmboe) and by AF's UK Medical Research Council Industrial Collaborative Awards in Science and Engineering (iCASE) studentship. The funder was not involved in the conceptualization, design, data collection, analysis, decision to publish, or preparation of the manuscript.

## CONFLICT OF INTEREST

The authors declare no conflicts of interest.

## ETHICS APPROVAL STATEMENT

The longitudinal study of which this study is part of received ethical approval from the University of Oxford Central University Research Ethics Committee: R57972/RE010.

## Supporting information


**Appendix S1** Supporting Information
**TABLE S1** Reasons questionnaires not sent
**TABLE S2** Missing data by assessment point
**TABLE S3** Groups of missing data
**TABLE S4** Patterns of missing data
**TABLE S5** Reported sample characteristics
**TABLE S6** Internal consistency (Cronbach's alpha) of questionnaire measures
**TABLE S7** Results of analyses using the surgency scale of the IBQ‐R‐VSF (Study 1)
**TABLE S8** Estimated marginal means for Models 1–3 with surgency as the dependent variable
**TABLE S9** Length of pandemic exposure (days) by sub‐sample
**TABLE S10** Descriptive statistics for ratings of MDS and infant temperament
**TABLE S11** Longitudinal stability of individual differences in MDS and temperament
**TABLE S12** Model 1: Assessment point and sub‐sample as fixed factors
**TABLE S13** Model 1: Estimated marginal means for MDS and temperament
**TABLE S14** Model 2: Assessment point and sub‐sample as fixed factors, MDS as a fixed covariate
**TABLE S15** Model 2: Estimated marginal means for temperament ratings (MDS as covariate)
**TABLE S16** Concurrent associations between MDS and infant temperament
**TABLE S17** Longitudinal associations between MDS and infant temperament
**TABLE S18** Sample characteristics
**TABLE S19** Descriptive statistics for the COVID‐19 stress score
**TABLE S20** Descriptive statistics for ratings of surgency on the IBQ‐R‐VSF
**TABLE S21** Concurrent associations between MDS and child temperament (controlling for age)
**TABLE S22** Longitudinal associations between MDS and child temperament (controlling for age)
**TABLE S23** Concurrent associations between COVID‐19 stress and child temperament (controlling for age)
**TABLE S24** Longitudinal associations between COVID‐19 stress and child temperament (controlling for age)
**FIGURE S1** Frequency histogram with normal distribution curve of the COVID‐19 stress score

## Data Availability

The data that support the findings of this study are available on the Open Science Framework website [https://osf.io/zg97d/] under a CC‐By Attribution 4.0 International licence.
