# Supplementary material for: Maternal depressive symptoms and early childhood temperament before and during the COVID‐19 pandemic in the United Kingdom
Source: Infant Child Dev. 2022 Jun 14:e2354. Online ahead of print. doi: 10.1002/icd.2354 (PMC9349650; doi:10.1002/icd.2354)
Supplement: Supplementary file 1 — Appendix S1 Supporting Information TABLE S1 Reasons questionnaires not sent TABLE S2 Missing data by assessment point TABLE S3 Groups of missing data TABLE S4 Patterns of missing data TABLE S5 Reported sample characteristics TABLE S6 Internal consistency (Cronbach's alpha) of questionnaire measures TABLE S7 Results of analyses using the surgency scale of the IBQ‐R‐VSF (Study 1) TABLE S8 Estimated marginal means for Models 1–3 with surgency as the dependent variable TABLE S9 Length of pandemic exposure (days) by sub‐sample TABLE S10 Descriptive statistics for ratings of MDS and infant temperament TABLE S11 Longitudinal stability of individual differences in MDS and temperament TABLE S12 Model 1: Assessment point and sub‐sample as fixed factors TABLE S13 Model 1: Estimated marginal means for MDS and temperament TABLE S14 Model 2: Assessment point and sub‐sample as fixed factors, MDS as a fixed covariate TABLE S15 Model 2: Estimated marginal means for temperament ratings (MDS as covariate) TABLE S16 Concurrent associations between MDS and infant temperament TABLE S17 Longitudinal associations between MDS and infant temperament TABLE S18 Sample characteristics TABLE S19 Descriptive statistics for the COVID‐19 stress score TABLE S20 Descriptive statistics for ratings of surgency on the IBQ‐R‐VSF TABLE S21 Concurrent associations between MDS and child temperament (controlling for age) TABLE S22 Longitudinal associations between MDS and child temperament (controlling for age) TABLE S23 Concurrent associations between COVID‐19 stress and child temperament (controlling for age) TABLE S24 Longitudinal associations between COVID‐19 stress and child temperament (controlling for age) FIGURE S1 Frequency histogram with normal distribution curve of the COVID‐19 stress score [file ICD-9999-0-s001.docx]

# **Contents**

[**Contents** 1](#_Toc96955595)

[**Supplementary Materials 1** 2](#_Toc96955596)

[**1.1.** **Context of the current study: COVID-19 national measures and restrictions in the UK in 2020-2021** 2](#_Toc96955597)

[**Supplementary Materials 2** 4](#_Toc96955598)

[**2.1. Questionnaire Administration and Response Rate (Study 1 & 2)** 4](#_Toc96955599)

[**2.2. Missing Data and Attrition (Study 1 & 2)** 4](#_Toc96955600)

[**Supplementary Materials 3** 7](#_Toc96955601)

[**3.1. Study 1 - Reported Sample Characteristics** 7](#_Toc96955602)

[**Supplementary Materials 4** 8](#_Toc96955603)

[**4.1. Internal Consistency of Questionnaire Measures** 8](#_Toc96955604)

[**Supplementary Materials 5** 9](#_Toc96955605)

[**5.1. Study 1: Results of Analyses involving the Surgency scale (IBQ-R-VSF)** 9](#_Toc96955606)

[**Supplementary Materials 6** 12](#_Toc96955607)

[**6.1. Study 1 - Length of Pandemic Exposure** 12](#_Toc96955608)

[**Supplementary Materials 7** 13](#_Toc96955609)

[**7.1. Study 1 - Additional Analyses, During Pandemic Sub-Sample as Separate Sample** 13](#_Toc96955610)

[**7.2. Longitudinal Stability** 14](#_Toc96955611)

[**7.3. Linear Mixed Models to Investigate Change across Assessment Points** 15](#_Toc96955612)

[**7.4. Longitudinal and Within-Age Associations between MDS and Temperament** 18](#_Toc96955613)

[**Supplementary Materials 8** 20](#_Toc96955614)

[**8.1.**  **Study 2 – Reported Sample Characteristics** 20](#_Toc96955615)

[**Supplementary Materials 9** 21](#_Toc96955616)

[**9.1.**  **Study 2 - COVID-19 Stress Scale** 21](#_Toc96955617)

[**Supplementary Materials 10** 22](#_Toc96955618)

[**10.1. Study 2 – Descriptive Statistics for the Surgency scale of the IBQ-R-VSF** 22](#_Toc96955619)

[**10.2. Study 2 - Linear Mixed Models with Data from the IBQ-R-VSF** 23](#_Toc96955620)

[**10.3.**  **Study 2 - Longitudinal and Concurrent Associations: Data from the IBQ-R-VSF** 23](#_Toc96955621)

[**Supplementary Materials 11** 26](#_Toc96955622)

[**11.1.**  **Study 2 – Concurrent and Longitudinal Associations between COVID-19 Stress and Temperament** 26](#_Toc96955623)

[**Supplementary Materials 12** 28](#_Toc96955624)

[**12.1.**  **Study 1 – Post-hoc Power Analyses** 28](#_Toc96955625)

[**12.2.**  **Study 2 – Post-hoc Power Analyses** 29](#_Toc96955626)

[**References for Supplementary Materials** 30](#_Toc96955627)

# **Supplementary Materials 1**

## **Context of the current study: COVID-19 national measures and restrictions in the UK in 2020-2021**

On January 30^th^, 2020, the World Health Organisation declared the COVID-19 pandemic a global health emergency. In March, the UK government encouraged the public to follow social distancing measures and maintain at least 2 metres separation from people outside of their household. On March 20^th^, all education and leisure facilities in the UK were ordered to close, and on March 23^rd^, the Prime Minister placed the country into a national lockdown under a “stay at home” order. Non-essential contact with people from outside the household was prohibited, and failure to comply with these measures could result in monetary police fines. The national lockdown restrictions were gradually eased from May 2020 and shops were permitted to open in June (with face masks made compulsory in shops in July). In the summer months, individuals could mix with other households indoors and were encouraged to “eat out to help out [the economy]” throughout August. Education facilities reopened in September. Individuals were still told to work from home where possible.

The UK saw an increase in COVID-19 cases in the autumn of 2020 and a series of local lockdowns and protective measures were introduced in areas with large numbers of cases (the so-called Tier System). A second national lockdown that would last for four weeks was announced on October 31^st^, 2020, in which education settings would remain open but all non-essential shops and leisure facilities would close. Non-essential contact with others from outside of the household was prohibited. In November, the county in which most participants in the study lived was placed into Tier 2 (‘high alert’); mixing of households was banned indoors, but up to six people could meet outside. Shops and restaurants remained open. Whilst the first COVID-19 vaccinations were administered in mid-December 2020, tougher local restrictions were enforced and many living in areas with high numbers of coronavirus cases were told that they could not mix with other households at Christmas. On Boxing Day, the local county entered Tier 4 (‘stay at home’) restrictions where individuals could only leave their house for essential purposes. It was also announced that a new UK variant of the coronavirus was more transmissible than the original virus, and that most new cases of COVID-19 in the country were likely caused by the mutated virus. On January 3^rd^, 2021, England entered its third national lockdown which included school closures and the return of the “stay at home” order. By the end of January 2021, the UK had more than 100,000 COVID-19 related deaths since the start of the pandemic out of a population of 66.65 million.

Source: <https://bfpg.co.uk/2020/04/covid-19-timeline/>.

# **Supplementary Materials 2**

## **2.1. Questionnaire Administration and Response Rate (Study 1 and 2)**

Response rates for the questionnaire packs sent at each assessment point are reported in the ‘Participants’ sections of the main manuscript for each study. Further information regarding the reasons why questionnaires were not sent to some participants at each assessment point are detailed in Table 1.

| Table 1 | |
| --- | --- |
| *Reasons Questionnaires Not Sent* | |
| Reason | *N* |
| Study 1: 10-months (Time 1) | |
| Joined study at 16-month assessment point | 4 |
| Withdrew from study | 2 |
| Total | 6 |
| Study 1: 16 months (Time 2) | |
| Did not respond to communications | 10 |
| Child illness – unable to participate in 16-month assessment point | 1 |
| Withdrew from study | 5 |
| 16-month assessment point still outstanding | 3 |
| Total | 19 |
| Study 2: April 2020 (Time 1) | |
| Total | 0 |
| Study 2: November 2020 (Time 2) | |
| Withdrew from study | 6 |
| Total | 6 |

## **2.2. Missing Data and Attrition (Study 1 and 2)**

Information about missing data by questionnaire type for each assessment point are provided in Table 2, and groups with missing data are reported in Table 3. Full details of the questionnaires used in both studies are provided in the Method sections of the main manuscript. Results from a missing value analysis (conducted in SPSS version 27) are reported in Table 4 and describe the pattern of missing data. Results of Little’s missing completely at random test (Little, 1988) suggest that the data in this study were missing completely at random for Study 1; χ^2^ (34) = 33.029, *p* = .515 and Study 2; χ^2^ (38) = 53.339, *p* = .050.

| Table 2 | | | | |
| --- | --- | --- | --- | --- |
| *Missing Data by Assessment Point* | | | | |
|  | **Study 1** | | **Study 2** | |
|  | 10 months | 16 months | April 2020 | November 2020 |
| N of questionnaires returned (%) | 173 / 181 (95.6%) | 144 / 168 (85.7) | 217 / 286  (75.9%) | 175 / 281  (62.3%) |
| N of partial responses | 17 | 15 | 14 | 0 |
| Missing Data | | | | |
| Beck Depression Inventory-II (Beck et al., 1996) | 16 / 173  (9.2%) | 40 / 168  (23.8%) | 74 / 286  (25.9%) | 106 / 281  (37.7%) |
| Infant Behavior Questionnaire (Revised, Very Short Form; Putnam et al., 2014) or Early Childhood Behavior Questionnaire (Very Short Form; Putnam et al., 2006) | 8 / 173  (4.6%) | 28 / 168  (16.7%) | 69 / 286  (24.1%) | 106 / 281  (37.7%) |
| COVID-19 Impact Questionnaire | **-** | **-** | 81 / 286  (28.3%) | 106 / 281  (37.7%) |

| Table 3 | |
| --- | --- |
| *Groups of Missing Data* | |
| Reason | *N* |
| Study 1: 10- and 16-months | |
| Joined the study at 16-month assessment point (no 10-month data) | 4 |
| 16-month assessment point upcoming (have not yet been sent questionnaire) | 7 |
| Missing 10-month BDI-II only | 6 |
| Missing 10-month IBQ-R-VSF only | 1 |
| Missing 10-month BDI-II and all 16-month data | 8 |
| Missing 10-month IBQ-R-VSF and all 16-month data | 1 |
| Missing all 10-month data and 16-month BDI-II | 5 |
| Missing 16-month BDI-II only | 12 |
| Missing 16-month IBQ-R-VSF only | 1 |
| Missing all 16-month data | 18 |
| Withdrew from study – no data | 7 |
| Total | 70 |
| Study 2: April and November 2020 |  |
| No response (all data missing) | 57 |
| No April data and were not sent November Questionnaire | 6 |
| Missing April Data | 7 |
| Missing November Data | 4 |
| Missing April COVID-19 Impact Questionnaire only | 2 |
| Missing April COVID-19 Impact and all November Data | 2 |
| Missing April BDI-II and April COVID-19 Impact Questionnaire | 1 |
| Missing April BDI-II, COVID-19 Impact Questionnaire and All November Data | 4 |
| Missing April COVID-19 Impact Questionnaire and all November Data | 3 |
| Withdrew from study – no data | 2 |
| Total | 88 |

| Table 4 | | |
| --- | --- | --- |
| *Patterns of Missing Data* | | |
| Type of Missing Data | **N** | **Patterns of Missing Data** |
| Study 1 |  |  |
| Missing MDS questionnaire at 10m | 6 | Including 1 participant with extreme high^*^ MDS score at 16-month assessment point |
| Missing MDS questionnaire at 16m | 13 | Questionnaire not completed |
| Missing MDS questionnaire at 10m and 16m | 1 | Questionnaires not completed |
| Missing all 10m data | 5 | Questionnaires not completed |
| Missing all 16m data | 29 | Including 4 participants who had extreme high^*^ MDS scores at 10-months |
|  |  | Including 1 participant had extreme low^*^ infant Negative Affect at 10-months |
| Study 2 | | |
| Missing COVID-19 Stress in April | 3 | Including 1 participant had extreme high^*^ MDS score in April & November |
| Missing MDS and COVID-19 Stress in April | 1 | Questionnaires not completed |
| Missing all April data | 8 | Questionnaires not completed |
| Missing all November data | 35 | Questionnaires not completed |
| Missing April COVID-19 Stress and all November Data | 7 | Questionnaires not completed |
| Missing April MDS and all November Data | 1 | Questionnaires not completed |
| Missing April MDS, April COVID-19 Stress, and all November Data | 4 | Questionnaires not completed |
| *Note.* Reported above are the results of a missing value analysis (missing data patterns) conducted in SPSS. All participants who were sent the questionnaires at each time point were included in the missing value analysis; Study 1: 10-months (*N* = 181), 16-months (*N* = 168), Study 2: April 2020 (*N* = 286), November 2020 (*N* = 281).  *‘*MDS questionnaire’ refers to the Beck Depression Inventory-II (see *Materials),* 10m = 10-months (Time 1), 16m = 16-months (Time 2), April = April 2020 (Time 1), November = November 2020 (Time 2).  ^*^ Extreme values are calculated as being +/- 1.5* the inter-quartile range. | | |

# **Supplementary Materials 3**

## **3.1. Study 1 - Reported Sample Characteristics**

Characteristics of the reported sample in Study 1 (*N* = 175) are presented in Table 5. Demographic information (background questionnaire) was collected when the child was 10-months of age. Six participants who were sent the background questionnaire did not complete it and so their data is unavailable. Some demographic information about the parents of a small number of participants were unavailable, as reflected in the *N.*

| Table 5 | | | | | | | |
| --- | --- | --- | --- | --- | --- | --- | --- |
| *Reported Sample Characteristics* | | | | | | | |
|  | ***N*** | | **Mean** | **SD** | **Minimum** | | **Maximum** |
| Child’s age when background questionnaire received (10-months) | 168 | | 9.68 | .48 | 9.11 | | 11.21 |
| Child’s age when background questionnaire received (if after 10-months) | 7 | | 16.84 | 3.96 | 12.27 | | 25.17 |
| Mother’s age | 174 | | 34.15 | 4.55 | 21 | | 53 |
| Mother’s years of education | 165 | | 17.91 | 3.10 | 10 | | 28 |
| Father’s age | 169 | | 35.55 | 5.53 | 22 | | 57 |
| Father’s years of education | 155 | | 17.11 | 3.30 | 9 | | 30 |
| Annual household income | 138 | | £76,343 | £31,565 | £18,000 | | £240,000 |
| Index of Multiple Deprivation Decile | 173 | | 7.47 | 2.20 | 2 | | 10 |
| Child’s Ethnicity | | ***N*** | | | | **%** | |
| Asian | | 5 | | | | 2.86 | |
| Mixed – Other | | 4 | | | | 2.29 | |
| Mixed White-Asian | | 7 | | | | 4.00 | |
| Mixed White-African/Caribbean | | 5 | | | | 2.86 | |
| Other Ethnic Group | | 2 | | | | 1.14 | |
| Other White | | 27 | | | | 15.43 | |
| White British | | 123 | | | | 70.29 | |
| Prefer not to say/did not answer | | 2 | | | | 0.57 | |
| Total | | 175 | | | | 100 | |

# **Supplementary Materials 4**

## **4.1. Internal Consistency of Questionnaire Measures**

The internal consistency (Cronbach’s alpha) for each of the questionnaire measures and sub-scales used in both Study 1 and 2 are detailed below in Table 6.

| Table 6 | | | | | | |
| --- | --- | --- | --- | --- | --- | --- |
| *Internal Consistency (Cronbach’s alpha) of Questionnaire Measures* | | | | | | |
| Questionnaire | **Scale** | **N of Items** | **Study 1** | | **Study 2** | |
|  |  |  | **10m** | **16m** | **April** | **November** |
| BDI-II | Depressive Symptoms | 21 | .93  *162* | .94  *134* | .92  *212* | .93  *175* |
| IBQ-R-VSF | Surgency | 13 | .48  *171* | .52  *145* | .46  *93* | ^*^ |
|  | Negative Affect | 12 | .75  *171* | .70  *146* | .73  *93* | .55  *17* |
|  | Orienting/Regulatory Capacity | 12 | .63  *171* | .63  *146* | .62  *93* | .66  *17* |
| ECBQ-VSF | Surgency | 12 |  |  | .66  *125* | .66  *158* |
|  | Negative Affect | 12 |  |  | .70  *125* | .65  *158* |
|  | Effortful Control | 12 |  |  | .72  *125* | .70  *158* |
| COVID-19 Impact Questionnaire | COVID-19 Stress Score | 10 |  |  | .85  *159* | .87  *159* |
| *Note.* Numbers in italics represent the *N* (number of data points contributing to the alpha).  ^*^ Cronbach’s alpha could not be calculated because there was a negative average covariance among items, which violates the assumptions of the reliability model. This is likely due to the small number of participants contributing data to this 13-item scale (*N* = 17). | | | | | | |

# **Supplementary Materials 5**

## **5.1. Study 1: Results of Analyses involving the Surgency scale (IBQ-R-VSF)**

Since the Cronbach’s alpha for the IBQ-R-VSF Surgency scale was too low to be considered reliable (α = .48), we do not include this scale in the analyses reported in Study 1 of the main manuscript. However, results will be reported here for purposes of transparency. Descriptive statistics and results of longitudinal stability correlations and within-age and longitudinal associations between Surgency and MDS are reported in Table 7 below.

| Table 7 | | | | |
| --- | --- | --- | --- | --- |
| *Results of Analyses using the Surgency Scale of the IBQ-R-VSF (Study 1)* | | | | |
|  | **Pre-pandemic** | | **Pre-to during pandemic** | |
|  | 10-months | 16-months | 10-months | 16-months |
| N | 78 | 71 | 89 | 67 |
| Mean (SD) | 4.97 (.64) | 5.17 (.60) | 4.97 (.63) | 5.12 (.57) |
| Within-age Associations: Surgency & MDS | *r* (70) = .172,  *p* = .149,  [-.065, .355] | *r* (60) = .202,  *p* = .115,  [-.084, .424] | *r* (79) = .022,  *p* = .846,  [-.197, .272] | *r* (58) = -.038,  *p* = .769,  [-.225, .200] |
| Longitudinal Stability of Surgency Ratings | *r* (63) = .557, *p* <.001,  [.369, .174] | | *r* (64) = .460, *p* <.001,  [.302, .607] | |
| 10-month MDS & 16m Surgency | *r* (60) = .228, *p* = .075,  [-.075, .448] | | *r* (60) = -.040, *p* = .758,  [-.076, .468] | |
| 10-month Surgency & 16m MDS | *r* (56) = .137, *p* = .304,  [-.139, .358] | | *r* (58) = .017, *p* = .899,  [-.232, .315] | |
| *Note.* MDS = Maternal Depressive Symptoms, as measured with the Beck Depression Inventory, second edition. | | | | |

**Linear Mixed Models**

To test whether ratings of Surgency significantly differed across assessment points, we conducted a fully factorial linear mixed model with Surgency as the dependent variable, participants as the random factor, and assessment point and sub-sample as fixed factors (Model 1). See Table 8 for estimated marginal means.

A significant effect of assessment point was found for ratings of Surgency; *F* (1, 151.204) = 12.817, *p* <.001. Pairwise comparisons revealed a significant increase in Surgency ratings from 10-months (*M* = 4.98, *SE* = .049) to 16-months (*M* = 5.16, *SE* = .048). No significant main effect of sub-sample; *F* (1, 173.471) = .271, *p* = .603, or interaction was found; *F* (1, 151.204) = .108, *p* = .743.

We also conducted a model that included MDS as a fixed covariate (Model 2, see Table 8). The effect of assessment point was still significant when including MDS as a fixed covariate; *F* (1, 138.982) = 6.795, *p* = .010. However, there was no significant main effect of MDS; *F* (1, 212.429) = 2.216, *p* = .138, or sub-sample; *F* (1, 186.293) = .994, *p* = .320. There were also no significant interactions between assessment point and MDS; *F* (1, 140.450) = .002, *p* = 969, assessment point and sub-sample; *F* (1, 138.982) = .069, *p* = .794, and sub-sample and MDS; *F* (1, 212.429) = 1.815, *p* = .179. The three-way interaction between assessment point, sub-sample, and MDS was also not significant; *F* (1, 140.450) = .411, *p* = .522.

To investigate whether the main effects of assessment point was because of pandemic exposure, a further model was conducted with length of pandemic exposure (days) as a fixed covariate (Model 3, see Table 8). A significant effect of assessment point was still found when considering pandemic exposure as a fixed covariate; *F* (1, 124.214) = 16.158, *p* <.001. There was no significant main effect of sub-sample; *F* (1, 129.029) = 1.634, *p* = .203, or of pandemic exposure; *F* (1, 127.761) = 1.674, *p* = .198. There was no significant assessment point and sub-sample interaction; *F* (1, 121.106) = .047, *p* = .828, and no significant assessment point and pandemic exposure interaction; *F* (1, 119.604) = .000, *p* = .983.

**Table 8**

*Estimated Marginal Means for Models 1 – 3 with Surgency as the Dependent Variable*

|  | Model 1 | Model 2 | Model 3 |
| --- | --- | --- | --- |
| Assessment Point |  |  |  |
| 10-months | 4.98 (.049) | 4.96 (.051) | 4.96 (.055) |
| 16-months | 5.16 (.048) | 5.15 (.052) | 5.18 (.051) |
| Mean Difference | .183 (.051) | .203 (.054) | .220 (.054) |
| Univariate Test of Assessment Point | ***F* (1, 151.204) = 12.817, *p* < .001** | ***F* (1, 132.800) = 14.241, *p* <.001** | ***F* (1, 124.214) = 16.158, *p* <.001** |
| Sub-Sample | |  |  |
| Pre-pandemic | 5.09 (.059) | 5.05 (.063) | 5.16 (.083) |
| Pre- to during pandemic | 5.05 (.058) | 5.05 (.061) | 4.95 (.107) |
| Mean Difference | .043 (.083) | .005 (.087) | .213 (.167) |
| Univariate Test of Sub-Sample | *F* (1, 173.471) = .271, *p* = .603 | *F* (1, 160.919) = .003, *p* = .954 | *F* (1, 129.029) = 1.634, *p* = .203 |
| *Note.* Estimated marginal means (standard error) are reported. MDS covariate is entered into the model as 9.6957. | | | |

# **Supplementary Materials 6**

## **6.1. Study 1 - Length of Pandemic Exposure**

Participants in the ‘pre- to during pandemic’ sub-sample (*N* = 70) contributed their 16-month questionnaire after the onset of the pandemic in the United Kingdom. There was also a small sub-sample of participants (*N* = 21) who contributed both their 10- and 16-month questionnaire data after the onset of the pandemic in the United Kingdom (‘during pandemic’ sub-sample). It is important to note that the length of pandemic exposure experienced varied for participants in these sub-samples. Table 9 contains descriptive statistics for the length of time (days) between the start of the first UK lockdown (23/03/2020) and receipt of the questionnaire packs.

| Table 9 | | | | | | |
| --- | --- | --- | --- | --- | --- | --- |
| *Length of Pandemic Exposure (Days) by Sub-Sample* | | | | | | |
|  |  |  | *N* | Mean | SD | Range |
| Pre- to during pandemic | 16-month |  | 56 | 95.68 | 49.57 | 8 - 185 |
| During pandemic | 10-month |  | 21 | 57.29 | 45.97 | 6 - 144 |
|  | 16-month |  | 12 | 207.33 | 22.81 | 162 - 246 |

*Note.* The pre- to during pandemic sample all submitted their 10-month questionnaires before the pandemic onset.

# **Supplementary Materials 7**

## **7.1. Study 1 - Additional Analyses, During Pandemic Sub-Sample as Separate Sample**

In our main analyses, the ‘during pandemic’ sub-sample was absorbed into the ‘pre- to during pandemic’ sub-sample. This is because the small size of the ‘during pandemic’ sample (*N* = 21) would limit our ability to make meaningful conclusions about the data provided by this group. We tested for convergence in results by conducting the analyses both with, and without, the ‘during pandemic’ group data, and these are reported below. Descriptive statistics for maternal depression and infant temperament ratings are presented in Table 10. Note that the results reported below include data from the Surgency scale of the IBQ-R-VSF, which we decided to exclude from the analyses reported in the main manuscript due to poor internal consistency. It is important to consider this when evaluating results pertaining to this scale.

| Table 10 | | | | | | | |
| --- | --- | --- | --- | --- | --- | --- | --- |
| *Descriptive Statistics for Ratings of MDS and Infant Temperament* | | | | | | | |
|  | *N* | Maternal Depressive Symptoms |  | *N* | Surgency | Negative Affect | Effortful Control |
| Pre-pandemic | | | | | | | |
| 10-months | 72 | 10.64 (9.31) | 78 | | 4.97 (.64) | 4.22 (.95) | 4.70 (.69) |
| 16-months | 62 | 9.90 (10.12) | 71 | | 5.17 (.60) | 4.28 (.75) | 5.01 (.64) |
| Pre- to during pandemic | | | | | | | |
| 10-months | 61 | 9.62 (9.11) | 68 | | 4.93 (.60) | 4.22 (.82) | 4.86 (.70) |
| 16-months | 52 | 8.21 (8.87) | 55 | | 5.15 (.55) | 4.08 (.87) | 5.13 (.60) |
| During pandemic | | | | | | | |
| 10-months | 20 | 9.30 (8.88) | 21 | | 5.08 (.75) | 3.81 (1.01) | 4.79 (.57) |
| 16-months | 9 | 10.67 (8.34) | 12 | | 5.00 (.67) | 4.30 (.65) | 5.26 (.52) |
| Pre- to during pandemic (incl. during pandemic) | | | | | | | |
| 10-months | 82 | 9.54 (9.00) |  | 89 | 4.97 (.63) | 4.13 (.88) | 4.84 (.67) |
| 16-months | 64 | 8.57 (8.77) |  | 67 | 5.12 (.57) | 4.12 (.84) | 5.15 (.60) |
| *Note.* Data in table represent mean, brackets contain standard deviation. *N* represents the number of responses that contribute to each statistic. | | | | | | | |

### **7.2. Longitudinal Stability**

Pearson’s correlation analyses indicated longitudinal stability of individual differences in MDS and infant temperament from 10- to 16-months (Table 11). All correlations remained significant when controlling the false discovery rate (Benjamini & Hochberg, 1995). Although we do not find evidence of longitudinal stability of individual differences in ratings of Negative Affect and Effortful Control in the ‘during pandemic’ sub-sample, this is likely a result of insufficient power to detect an effect (36% power to detect a large effect: *r* = .50). Overall, effect sizes are largely consistent across groups.

| Table 11 | | | | |
| --- | --- | --- | --- | --- |
| *Longitudinal Stability of Individual Differences in MDS and Temperament* | | | | |
|  | Maternal Depressive Symptoms | Surgency | Negative Affect | Effortful Control |
| Pre-pandemic | ***r* (53) = .872,**  ***p* <.001,**  **[.734, .937]** | ***r* (63) = .557,**  ***p* <.001,**  **[.369, .174]** | ***r* (63) = .376,**  ***p* = .002,**  **[.170, .558]** | ***r* (63) = .506,**  ***p* <.001,**  **[.250, .685]** |
| Pre- to during pandemic | ***r* (46) = .858,**  ***p* <.001,**  **[.618, .949]** | ***r* (52) = .428,**  ***p* <.001,**  **[.254, .610]** | ***r* (52) = .364,**  ***p* = .007,**  **[121, .571]** | ***r* (52) = .662,**  ***p* <.001,**  **[.467, .790]** |
| During pandemic | ***r* (7) = .915,**  ***p* < .001,**  **[.207, .985]** | ***r* (10) = .618,**  ***p* = .032,**  **[.268, .856]** | *r* (10) = .368,  *p* = .239,  [-.237, .916] | *r* (10) = .443,  *p* = .150,  [-.152, .848] |
| Pre- to during pandemic (incl. during) | ***r* (55) = .861,**  ***p* = <.001,**  **[.661, .948]** | ***r* (64) = .460,**  ***p* <.001,**  **[.302, .607]** | ***r* (64) = .338,**  ***p* = .005,**  **[.109, .549]** | ***r* (64) = .633,**  ***p* <.001,**  **[.469, .766]** |
| *Note.* Square brackets represent 95% confidence intervals which were calculated in SPSS on 1000 bootstrap samples. | | | | |

### **7.3. Linear Mixed Models to Investigate Change across Assessment Points**

To investigate changes in ratings of MDS and temperament from 10- to 16- months, linear mixed models with assessment point (2 levels) and sub-sample (2 levels) as fixed factors and participants as a random factor were conducted. The sub-samples included in this model were the pre-pandemic sub-sample, and the pre- to during pandemic sub-sample that does not include the small sample of participants with during pandemic data at both 10- and 16-months. Results are consistent with the effect sizes reported in the main manuscript when the during-pandemic group are absorbed into the pre- to during pandemic group.

In Table 12, we report the Type III Fixed Effects of the models that included assessment point and sub-sample as fixed factors, and participants as the random factor (Model 1). Estimated marginal means and results of the univariate *F*-test of assessment point for Model 1 are reported in Table 13. Table 14 displays the Type III Fixed Effects of the models that included assessment point and sub-sample as fixed factors, participants as the random factor, and MDS as the fixed covariate (Model 2). Table 15 displays the estimated marginal means and univariate tests for Model 2.

**Results**

*MDS.* There was no significant effect of assessment point or sub-sample.

*Negative Affect.* There was no significant effect of assessment point or sub-sample on ratings of Negative Affect, nor any significant effect of MDS on ratings of Negative Affect. There was a significant assessment point × MDS interaction for Negative Affect; *t* (129.496) = 2.575, *p* = .011. This is because MDS and Negative Affect were significantly correlated at 10-months, but not at 16-months.

*Surgency.* Results indicated a significant increase in ratings of Surgency from 10-months (*M* = 4.96, *SE* = .051) to 16-months (*M* = 5.17, *SE* = .051); *F* (1, 133.588) = 16.490, *p* <.001. There was no significant effect of sub-sample, and no significant main effects of interactions with MDS.

*Effortful Control.* Ratings of Effortful Control were significantly higher at 16-months (*M* = 5.07, *SE* = .054) than at 10-months (*M* = 4.78, *SE* = .058); *F* (1, 128.555) = 30.344, *p* <.001. There was no significant effect of sub-sample, and no significant influence of MDS on ratings of Effortful Control.

| Table 12 | | | | |
| --- | --- | --- | --- | --- |
| *Model 1: Assessment Point and Sub-Sample as Fixed Factors* | | | | |
|  | MDS | Surgency | Negative Affect | Effortful Control |
| Assessment Point | *F* (1, 107.023) = 3.278, *p =* .073 | ***F* (1, 133.588) = 16.490, *p* <.001** | *F* (1, 137.453) = .084, *p* = .772 | ***F* (1, 128.555) = 30.344, *p* <.001** |
| Sub-Sample | *F* (1, 143.787) = 1.296, *p* = .257 | *F* (1, 151.166) = .305, *p* = .582 | *F* (1, 149.746) = .770, *p* = .382 | *F* (1, 147.690) = 1.848, *p* = .176 |
| Assessment Point x Sub-Sample | *F* (1, 107.023) = 2.424, *p* = .122 | *F* (1, 133.588) = .077, *p* = .782 | *F* (1, 137.453) = .850, *p* = .358 | *F* (1, 128.555) = .032, *p* = .858 |

**Table 13**

*Model 1: Estimated Marginal Means for MDS and Temperament*

|  | MDS | Surgency | Negative Affect | Effortful Control |
| --- | --- | --- | --- | --- |
| Assessment Point | | | | |
| 10-months | 10.03 (.779) | 4.96 (.051) | 4.23 (.084) | 4.78 (.058) |
| 16-months | 9.15 (.816) | 5.17 (.051) | 4.20 (.071) | 5.07 (.054) |
| Mean Difference | .855 (.472) | .215 (.053) | .024 (.084) | .295 (.054) |
| Univariate Test of Assessment Point | *F* (1, 107.023) = 3.278, *p* = .073 | ***F* (1, 133.588) = 16.490, *p* < .001** | *F* (1, 137.453) = .084, *p* = .772 | ***F* (1, 128.555) = 30.344, *p* < .001** |
| Sub-Sample | | | | |
| Pre-pandemic | 10.44 (1.024) | 5.09 (.058) | 4.27 (.079) | 4.86 (.066) |
| Pre- to during pandemic | 8.71 (1.128) | 5.04 (.064) | 4.16 (.087) | 4.99 (.073) |
| Mean Difference | 1.734 (1.523) | .048 (.087) | .103 (.117) | .134 (.098) |
| Univariate Test of Sub-Sample | *F* (1, 143.787) = 1.296, *p* = .257 | *F* (1, 151.166) = .305, *p* = .582 | *F* (1, 149.746) = .770, *p* = .382 | *F* (1, 147.690) = 1.848, *p* = .176 |
| *Note.* Estimated marginal means (standard error) are reported. | | | | |

| Table 14 | | | |
| --- | --- | --- | --- |
| *Model 2: Assessment Point and Sub-Sample as Fixed Factors, MDS as a Fixed Covariate* | | | |
|  | Surgency | Negative Affect | Effortful Control |
| Assessment Point | ***F* (1, 124.828) = 7.350, *p* = .008** | ***F* (1, 134.327) = 5.235, *p* = .024** | ***F* (1, 121.376) = 6.046, *p* = .015** |
| MDS | *F* (1, 186.026) = 2.317, *p* = .130 | *F* (1, 173.330) = 1.258, *p* = .264 | *F* (1, 193.403) = 1.145, *p* = .286 |
| Assessment Point x MDS | *F* (1, 125.214) = .005, *p* = .946 | ***F* (1, 135.707) = 9.191, *p* = .003** | *F* (1, 121.297) = 1.294, *p* = .258 |
| Sub-Sample | *F* (1, 162.711) = .759, *p* = .385 | *F* (1, 158.117) = .885, *p* = .348 | *F* (1, 164.568) = .895, *p* = .346 |
| Assessment Point x Sub-Sample | *F* (1, 124.828) = .197, *p* = .658 | *F* (1, 134.327) = .099, *p* = .753 | *F* (1, 121.297) = .986, *p* = .323 |
| Sub-Sample x MDS | *F* (1, 186.026) = 1.443, *p* = .231 | *F* (1, 173.330) = .268, *p* = .605 | *F* (1, 193.403) = .004, *p* = .950 |
| Assessment Point x Sub-Sample x MDS | *F* (1, 125.214) = .247, *p* = .620 | *F* (1, 135.707) = .753, *p* = .387 | *F* (1, 121.297) = .512, *p* = .476 |

**Table 15**

*Model 2: Estimated Marginal Means for Temperament Ratings (MDS as Covariate)*

|  | Surgency | Negative Affect | Effortful Control |
| --- | --- | --- | --- |
| Assessment Point | | | |
| 10-months | 4.93 (.053) | 4.19 (.074) | 4.77 (.058) |
| 16-months | 5.16 (.054) | 4.20 (.074) | 5.04 (.056) |
| Mean Difference | .226 (.057) | .008 (.088) | .273 (.057) |
| Univariate Test of Assessment Point | ***F* (1, 120.629) = 15.951, *p* <.001** | *F* (1, 128.008) = .008, *p* = .930 | ***F* (1, 118.080) = 22.948, *p* <.001** |
| Sub-Sample | | | |
| Pre-pandemic | 5.05 (.061) | 4.24 (.080) | 4.83 (.067) |
| Pre- to during pandemic | 5.05 (.067) | 4.15 (.088) | 4.97 (.073) |
| Mean Difference | .002 (.091) | .095 (.119) | .136 (.099) |
| Univariate Test of Sub-Sample | *F* (1, 140.888) = .000, *p* = .987 | *F* (1, 142.076) = .638, *p* = .426 | *F* (1, 139.422) = 1.885, *p* = .172 |

*Note.* Mean (standard error) are reported for each of the estimated marginal means. MDS covariate is entered into the model as 9.6923.

To investigate whether the main effects of assessment point for Surgency and Effortful Control were associated with pandemic exposure, two further linear mixed models were conducted with length of pandemic exposure (days) as a fixed covariate. Since earlier models revealed no significant change in ratings of Negative Affect across assessment points, we did not conduct these analyses on this variable. There were no significant main effects of pandemic exposure in ratings of Surgency; *F* (1, 252.826) = 2.516, *p* = .114, and Effortful Control; *F* (1, 251.342) = 2.451, *p* = .119, nor interactions associated with pandemic exposure (*p* > .05). Ratings of Surgency and Effortful Control both significantly (*p* <.001) increased across assessment points; however, this increase was unaffected by length of pandemic exposure.

### **7.4. Longitudinal and Within-Age Associations between MDS and Temperament**

Pearson correlation analyses were conducted to investigate concurrent associations between MDS and infant temperament (Table 16). Results across sub-samples appear to converge in most cases, although whereas there is a significant association between MDS and Negative Affect at 10-months in the ‘pre to during’ group, there is no such association in the ‘during pandemic’ group. Again, this is likely due to a lack of power in the small ‘during pandemic’ group. However, when the groups are combined this association is significant. All correlations remained significant when controlling the false discovery rate.

| Table 16 | | | |
| --- | --- | --- | --- |
| *Concurrent Associations between MDS and Infant Temperament* | | | |
|  | MDS & Surgency | MDS & Negative Affect | MDS & Effortful Control |
| 10 months |  |  |  |
| Pre-pandemic | *r* (70) = .172, *p* = .149, [-.065, .355] | *r* (70) = .170, *p* = .154, [-.037, .345] | *r* (70) = -.216, *p* = .089, [-.375, -.065] |
| Pre- to during pandemic | *r* (59) = .029, *p* = .827, [-.245, .362] | ***r* (59) = .375, *p* = .003, [.173, .620]** | *r* (59) = -.026, *p* = .842, [-.188, .159] |
| During pandemic | *r* (18) = .008, *p* = .972, [-.421, .387] | *r* (18) = .315, *p* = .176, [-.025, .605] | *r* (18) = -.126, *p* = .597, [-.624, .354] |
| Pre- to during pandemic (incl. during) | *r* (79) = .022, *p* = .846, [-.197, .272] | ***r* (79) = .353, *p* = .001, [.183, .527]** | *r* (79) = -.046, *p* = .683, [-.227, .121] |
| 16 months |  |  |  |
| Pre-pandemic | *r* (60) = .202, *p* = .115, [-.084, .424] | *r* (60) = -.133, *p* = .302, [-.385, .192] | *r* (60) = -.080, *p* = .536, [-.279, .136] |
| Pre- to during pandemic | *r* (50) = -.035, *p* = .804, [-.251, .233] | *r* (50) = -.117, *p* = .408, [-.343, .111] | *r* (50) = -.060, *p* = .675, [-.435, .182] |
| During pandemic | *r* (7) = -.034, *p* = .932, [-.665, .552] | *r* (7) = -.401, *p* = .284, [-.836, .948] | *r* (7) = .315, *p* = .410, [-.639, .816] |
| Pre- to during pandemic (incl. during) | *r* (58) = -.038, *p* = .769, [-.225, .200] | *r* (58) = -.133, *p* = .308, [-.359, .101] | *r* (58) = -.012, *p* = .927, [-.332, .248] |
| *Note.* 95% Confidence intervals are reported in square brackets. | | | |

Further correlational analyses revealed no significant longitudinal associations between 10-month MDS and 16-month infant temperament, or 10-month temperament and 16-month MDS in either group (Table 17). Again, this mirrors the results reported in the main manuscript when the ‘during’ group were included in the ‘pre- to during’ sub-sample.

| Table 17 | | | | | | |
| --- | --- | --- | --- | --- | --- | --- |
| *Longitudinal Associations between MDS and Infant Temperament* | | | | | | |
|  | **10-months MDS and 16-month Infant Temperament** | | | **10-month Infant Temperament and 16-month MDS** | | |
|  | MDS & Surgency | MDS & Negative Affect | MDS & Effortful Control | Surgency & MDS | Negative Affect & MDS | Effortful Control & MDS |
| Pre-pandemic | *r* (60) = .228, *p* = .075, [-.075, .448] | *r* (60) = .094, *p* = .468, [-.160, .363] | *r* (60) =  -.151, *p* = .241, [-.345, .064] | *r* (56) = .137, *p* = .304, [-.139, .358] | *r* (56) =  -.041, *p* = .760, [-285, 193] | *r* (56) = -.244, *p* = .064,  [-.452,  -.069] |
| Pre- to during pandemic | *r* (49) =  -.039, *p* = .788, [-.227, .220] | *r* (49) = .047, *p* = .743, [-.149, .283] | *r* (49) = .100, *p* = .483, [-.208, .305] | *r* (49) =  -.016, *p* = .910, [-.278, .315] | *r* (49) = .153, *p* = .283, [-.117, .392] | *r* (49) = -.074, *p* = .605, [-.275, .151] |
| During pandemic | *r* (9) =  -.064, *p* = .852, [-.713, .662] | *r* (9) =  -.403, *p* = .219, [-.823, .733] | *r* (9) = .337, *p* = .311, [-.492, .750] | *r* (7) = .082, *p* = .835, [-.702, .671] | *r* (7) = .592, *p* = .093, [.245, .966] | *r* (7) = -.596, *p* = .090, [-.925, -.295] |
| Pre to during pandemic (incl. during) | *r* (60) =  -.040, *p* = .758, [-.076, .468] | *r* (60) =  -.001, *p* = .993, [-.202, .224] | *r* (60) = .129, *p* = .316, [-.148, .328] | *r* (58) = .017, *p* = .899, [-.232, .315] | *r* (58) = .213, *p* = .102, [.015, .397] | *r* (58) = -.132, *p* = .314, [-.323, .049] |
| *Note*. 95% Confidence intervals are reported in square brackets | | | | | | |

# **Supplementary Materials 8**

## **8.1. Study 2 – Reported Sample Characteristics**

Characteristics of the reported sample in Study 2 (*N* = 220) are presented in Table 18. Demographic information for all participants involved in the Oxford Early EF study (*N* = 154) and in cross-sectional piloting for this study (*N* = 66) were collected using an online questionnaire that parents completed when the child was 10-months of age (longitudinal sample) or before their pilot testing session (pilot sample), which was also commonly at 10-months. Six participants from the Oxford Early EF sample were not sent the background questionnaire at 10-months (see SM 2.1), and 6 participants who were sent the background questionnaire did not complete it and so their data is unavailable.

**Table 18**

*Sample Characteristics*

|  | *N* | | Mean | SD | Minimum | | Maximum |
| --- | --- | --- | --- | --- | --- | --- | --- |
| Child’s age (months) when April questionnaire sent | 220 | | 19.63 | 8.16 | 5.00 | | 41.00 |
| Mother’s age | 201 | | 34.28 | 4.81 | 23 | | 64 |
| Mother’s years of education | 190 | | 18.26 | 3.03 | 10 | | 28 |
| Father’s age | 196 | | 35.79 | 5.83 | 22 | | 64 |
| Father’s years of education | 182 | | 17.41 | 3.28 | 9 | | 25 |
| Annual household income | 168 | | £76,014 | £35,477 | £18,000 | | £300,000 |
| Index of Multiple Deprivation Decile | 212 | | 7.6 | 2.12 | 2 | | 10 |
| Child’s Ethnicity | | ***n*** | | | | **%** | |
| Asian | | 3 | | | | 1.4 | |
| Mixed | | 3 | | | | 1.4 | |
| Mixed – Other | | 6 | | | | 2.7 | |
| Mixed White-Asian | | 8 | | | | 3.6 | |
| Mixed White-African/Caribbean | | 3 | | | | 1.4 | |
| Other Ethnic Group | | 2 | | | | 0.9 | |
| Other White | | 33 | | | | 15.0 | |
| White British | | 141 | | | | 64.1 | |
| Prefer not to say | | 20 | | | | 9.1 | |
| Did not answer | | 1 | | | | 0.5 | |
| Total | | 220 | | | | 100 | |

# **Supplementary Materials 9**

## **9.1. Study 2 - COVID-19 Stress Scale**

A measure of COVID-19 related stress was derived from ten questions asked in the COVID-19 Impact Questionnaire (<https://osf.io/zg97d/>). These questions (detailed in the ‘*Materials’* section for Study 2 in the main manuscript) captured the respondent’s experiences of negative emotions felt because of the pandemic as well as their perceptions about the impact of the pandemic. A COVID-19 stress score was created for each participant by summing the scores for each item; a larger score indicates a more stressful experience. Descriptive statistics are reported in Table 19. Distributions of the COVID-19 stress score for participants who contributed data in both April and November are presented in Figure 1.

**Table 19**

*Descriptive Statistics for the COVID-19 Stress Score*

|  | N | Mean | SD | Minimum | Maximum |
| --- | --- | --- | --- | --- | --- |
| April 2020 | 197 | 14.85 | 6.29 | 1 | 31 |
| November 2020 | 173 | 14.07 | 6.82 | 0 | 33 |

| **Figure 1** |
| --- |
| *Frequency Histogram with Normal Distribution Curve of the COVID-19 Stress Score* |
| 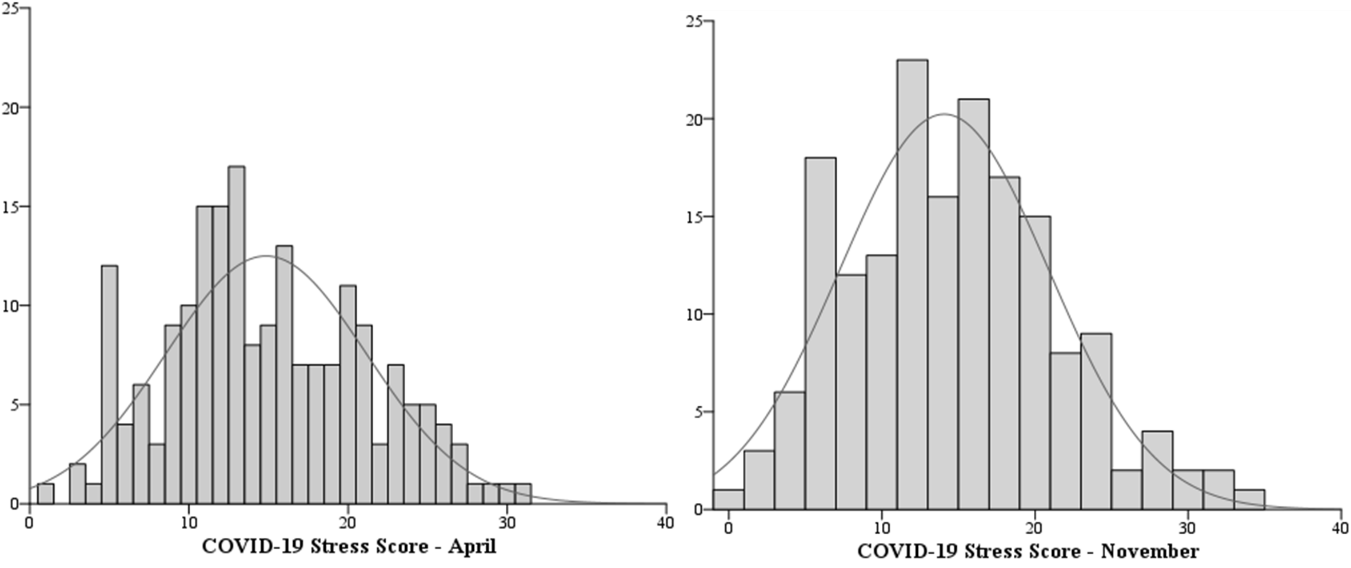 |

# **Supplementary Materials 10**

As the analysis section of Study 2 in the manuscript only included data from participants who completed the ECBQ-VSF at both assessment points, we will report data from the group who completed the IBQ-R-VSF at both assessment points below. For transparency, this includes data from the Surgency scale, which we decided to exclude from the analyses due to poor internal consistency. It is important to consider this when evaluating results pertaining to this scale. Although there were a sample of participants who completed the IBQ-R-VSF in April and ECBQ-VSF in November, it is not meaningful to conduct analyses when a different questionnaire was used across assessment points. This is because although they are related, the questionnaires do not contain any overlapping items. It would therefore be difficult to tease apart results that were purely a result of the change in measure from actual change over time. However, data from this group collected using the IBQ-R-VSF in April will be used when assessing concurrent correlations between temperament and MDS.

## **10.1. Study 2 – Descriptive Statistics for the Surgency scale of the IBQ-R-VSF**

Descriptive statistics for the IBQ-R-VSF are reported in the ‘Results’ section for Study 2 in the manuscript, however the Surgency scale was not included due to the poor internal consistency and so is reported in Table 20 below.

| **Table 20** | | | | | |
| --- | --- | --- | --- | --- | --- |
| *Descriptive Statistics for Ratings of Surgency on the IBQ-R-VSF* | | | | | |
|  | ***N*** | **IBQ-R-VSF** | ***N*** | ***N*** | **IBQ-R-VSF and ECBQ-VSF** |
| April | 21 | 4.84 (.72) | 119 | 71 | 5.04 (.53) |
| November | 17 | 4.90 (.46) | 96 | 60 | - |
| *Note.* Data in the final column are from the group who completed the IBQ-R-VSF in April 2020, and the ECBQ-VSF in November 2020. Analyses in this section of the report are focused only on the data collected with the IBQ-R-VSF, and so we only report the Surgency score from the IBQ-R-VSF in April 2020 for this group. | | | | | |

## **10.2. Study 2 - Linear Mixed Models with Data from the IBQ-R-VSF**

To assess change across assessment points in ratings of MDS, a linear mixed model was conducted with assessment point as a fixed factor and participants as the random factor (Model 1). Maximum likelihood estimation was employed to account for missing data, and a heterogeneous first-order autoregressive covariance structure was assumed due to the small sample size. Results indicated no significant change across assessment points (*p* = .997) from April to November.

Similar models were constructed to assess change in ratings of temperament across assessment points, when MDS and age (days) in April were entered as fixed covariates (Model 2). The models investigated the full factorial effects. Results indicated no significant (*p* >.05) effects or interactions involving MDS or age in any temperament dimension. There was also no significant change in ratings of Surgency (*p* = .725), Negative Affect (*p* = .923) or Effortful Control (*p* = .219) across assessment points when MDS and age were considered.

When COVID-19 related stress was entered into the model as a fixed covariate in addition to age (Model 3), results similarly indicated no significant effects or interactions in any temperament dimensions (*p* > .05). This suggests that there was no influence of COVID-19 related stress on ratings of temperament across assessment points.

## **10.3. Study 2 - Longitudinal and Concurrent Associations: Data from the IBQ-R-VSF**

Results of partial correlation analyses controlling for age indicated moderate stability of individual differences in ratings of Surgency across assessment points; *r* (14) = .497, *p* = .050, *CI* = [.101, .850], although this did not cross the significance threshold. It is important to consider the poor internal consistency of the Surgency scale and limited sample size when interpreting this marginal effect. There was also significant longitudinal stability for ratings of Effortful Control across assessment points; *r* (14) = .589, *p* = .016, *CI* = [.019, .884], but no stability in ratings of Negative Affect; *r* (14) = .431, *p* = .096, *CI* = [-.112, .748].

Within each assessment point, partial correlation analyses were conducted to investigate concurrent associations between MDS and child temperament when controlling for age (days) in April. Results (Table 21) indicated no within-assessment point associations between MDS and child temperament in April or November.

| Table 21 | |
| --- | --- |
| *Concurrent Associations between MDS and Child Temperament (Controlling for Age)* | |
| April |  |
| MDS & Surgency | *r* (88) = .093, *p* = .381, [-.139, .290] |
| MDS & Negative Affect | *r* (88) = .052, *p* = .629, [-.137, .256] |
| MDS & Effortful Control | *r* (88) = -.135, *p* = .203, [-.311, .021] |
| November |  |
| MDS & Surgency | *r* (14) = .167, *p* = .536, [-.322, .643] |
| MDS & Negative Affect | *r* (14) = -.090, *p* = .740, [-.658, .316] |
| MDS & Effortful Control | *r* (14) = -.010, *p* = .972, [-.429, .372] |
| *Note.* 95% confidence intervals are reported in square brackets. | |

To investigate potential longitudinal associations between MDS and temperament, partial correlational analyses were conducted across assessment points, controlling for age (Table 22). No longitudinal associations were found in the participants who completed the IBQ-R-VSF in April and November.

| Table 22 | |
| --- | --- |
| *Longitudinal Associations between MDS and Child Temperament (Controlling for Age)* | |
| April MDS & November Temperament | IBQ-R-VSF |
| MDS and Surgency | *r* (14) = -.007, *p* = .978,  [-.385, .469] |
| MDS and Negative Affect | *r* (14) = -.052, *p* = .848,  [-.715, .429] |
| MDS and Effortful Control | *r* (14) = -.097, *p* = .721,  [-.577, .365] |
| April Temperament & November MDS |  |
| Surgency and MDS | *r* (73) = .068, *p* = .562,  [-.189, .309] |
| Negative Affect and MDS | *r* (73) = .022, *p* = .854,  [-.221, .283] |
| Effortful Control and MDS | *r* (73) = -.059, *p* = .615,  [-.267, .120] |
| *Note.* 95% confidence intervals are reported in square brackets. | |
|  | |

# **Supplementary Materials 11**

## **11.1. Study 2 – Concurrent and Longitudinal Associations between COVID-19 Stress and Temperament**

Partial correlation analyses were conducted to investigate concurrent associations between COVID-19 stress and child temperament when controlling for age (days) in April (Table 23). Similar analyses were conducted to investigate potential longitudinal associations between COVID-19 stress and temperament when controlling for age (Table 24). No significant associations were found.

| Table 23 | | |
| --- | --- | --- |
| *Concurrent Associations between COVID-19 Stress and Child Temperament (Controlling for Age)* | | |
|  | **IBQ-R-VSF** | **ECBQ-VSF** |
| April |  |  |
| COVID-19 Stress & Surgency | *r* (84) = -.071, *p* = .516,  [-.277, .144] ^a^ | *r* (107) = .154, *p* = .110,  [-.052, .340] |
| COVID-19 Stress & Negative Affect | *r* (84) = -.009, *p* = .933,  [-.204, .173] | *r* (107) = -.012, *p* = .901,  [-.214, .193] |
| COVID-19 Stress & Effortful Control | *r* (84) = -.058, *p* = .594,  [-.256, .151] | *r* (107) = -.156, *p* = .104,  [-.322, .031] |
| November |  |  |
| COVID-19 Stress & Surgency | *r* (14) = .254, *p* = .343,  [-.364, .780] ^a^ | *r* (145) = .101, *p* = .225,  [-.039, .234] |
| COVID-19 Stress & Negative Affect | *r* (14) = -.090, *p* = .739,  [-.657, .474] | *r* (145) = .075, *p* = .369,  [-.089, .247] |
| COVID-19 Stress & Effortful Control | *r* (14) = -.144, *p* = .595,  [-.657, .300] | *r* (145) = -.119, *p* = .150,  [-.258, .023] |
| *Note.* 95% confidence intervals are reported in square brackets.  ^a^ Results should be interpreted with caution due to the poor internal consistency of the IBQ-R-VSF Surgency scale in this study. | | |

| Table 24 | | | |
| --- | --- | --- | --- |
| *Longitudinal Associations between COVID-19 stress and Child Temperament (Controlling for Age)* | | | |
|  | IBQ-R-VSF | ECBQ-VSF |  |
| April COVID-19 Stress & November Temperament | | | |
| COVID-19 Stress and Surgency | *r* (14) = .099, *p* = .716,  [-.477, .546] ^a^ | *r* (141) = .152, *p* = .070,  [-.015, .306] |  |
| COVID-19 Stress and Negative Affect | *r* (14) = -.059, *p* = .828,  [-.630, .410] | *r* (141) = -.024, *p* = .774,  [-.168, .142] |  |
| COVID-19 Stress and Effortful Control | *r* (14) = -.307, *p* = .248,  [-.704, .283] | *r* (141) = -.101, *p* = .232,  [-.256, .059] |  |
| April Temperament & November COVID-19 Stress | | |  |
| Surgency and COVID-19 Stress | *r* (73)= -.016, *p* = .889,  [-.204, .164] ^a^ | *r* (86) = .132, *p* = .221,  [-.103, .355] |  |
| Negative Affect and COVID-19 Stress | *r* (73) = -.074, *p* = .527,  [-.341, .206] | *r* (86) = .059, *p* = .584,  [-.130, .249] |  |
| Effortful Control and COVID-19 Stress | *r* (73) = .078, *p* = .508,  [-.111, .258] | *r* (86) = -.086, *p* = .428,  [-.303, .129] |  |
| *Note.* 95% confidence intervals are reported in square brackets.  ^a^ Results should be interpreted with caution due to the poor internal consistency of the IBQ-R-VSF Surgency scale in this study. | | | |

# **Supplementary Materials 12**

## **12.1. Study 1 – Post-hoc Power Analyses**

Post-hoc power analyses were conducted in G*Power (Faul et al., 2008) to investigate the minimum effect size that we were able to detect with each analysis.

*Correlational Analyses*

With a mean sample size of *N* = 63 (with the pre-pandemic group offering the smallest sample size for our correlations), a post-hoc power analysis revealed that the observed statistical power for correlational analyses with small effect sizes (*r* = .10) was 12.3%, medium effect sizes (*r* = .30) was 69%, and large effect sizes (*r* = .50) was 99%. Mean sample size was used here as the sample sizes of the correlations change depending on which variables are used in the analyses (because not all participants completed all questionnaires at all time points). In summary, our samples of *N* = 63 (mean; pre-pandemic group) and *N* = 65 participants (mean; pre-to-during pandemic group) were sufficiently powered to identify large effect sizes in correlational analyses.

Since we did not find any effects of sub-group (i.e., pandemic context), when we combine sub-samples (N = 124) for correlational analyses between MDS and Negative Affect, we have sufficient power (81%) to detect modest effects (*r* = .25). Whilst we do not have sufficient power to detect subtle pandemic effects in our individual sub-samples, we are well-powered to detect relatively modest general associations between MDS and temperament.

*Linear Mixed Models; Tests of Fixed Effects*

With a minimum sample size of *N* = 135 the observed statistical power for *F*-tests of fixed effects with small effect sizes (Cohen’s *f* = .10) was 21.09%, for medium effect sizes (Cohen’s *f* = .25) was 89.10%, and for large effect sizes (Cohen’s *f* = .40) was 99.91%. This suggests that our sample was well-powered to detect medium effect sizes when examining fixed effects within our linear mixed model analyses.

## **12.2. Study 2 – Post-hoc Power Analyses**

*Correlations*

With a mean sample size of *N* = 118, a post-hoc power analysis revealed that the observed statistical power for correlational analyses with small effect sizes (*r* = .10) was 19.14%, medium effect sizes (*r* = .30) was 92.33%, and large effect sizes (*r* = .50) was 99.99%. Mean sample size was used here because the sample sizes of the correlations change depending on which variables are used in the analyses (because not all participants completed all questionnaires at all time points). In summary, our samples were well-powered to identify medium and large effect sizes in correlational analyses.

*Linear mixed models*

With a minimum sample size of *N* = 113, the observed statistical power for *F*-tests of fixed effects with small effect sizes (Cohen’s *f* = .10) was 18.37%, with medium effect sizes (Cohen’s *f* = .25) was 74.99%, and with large effect sizes (Cohen’s *f* = .40) was 98.79%. This suggests that our sample was sufficiently powered to detect medium-to-large effect sizes when examining fixed effects within our linear mixed model analyses.

# **References for Supplementary Materials**

Beck, A., Steer, R., & Brown, G. (1996). *Beck Depression Inventory-II*. APA PsycTests. https://doi.org/10.1037/t00742-000

Benjamini, Y., & Hochberg, Y. (1995). Controlling the False Discovery Rate : A Practical and Powerful Approach to Multiple Testing. *Journal of the Royal Statistical Society*, *57*(1), 289–300. https://doi.org/10.1111/j.2517-6161.1995.tb02031.x

Little, R. J. (1988). A test of missing completely at random for multivariate data with missing values. *Journal of the American statistical Association*, *83*(404), 1198-1202.

Putnam, S. P., Gartstein, M. A., & Rothbart, M. K. (2006). Measurement of fine-grained aspects of toddler temperament: The Early Childhood Behavior Questionnaire. *Infant Behavior & Development*, *29*(3), 386–401. https://doi.org/10.1016/j.infbeh.2006.01.004

Putnam, S. P., Helbig, A. L., Gartstein, M. A., Rothbart, M. K., & Leerkes, E. (2014). Development and assessment of short and very short forms of the Infant Behavior Questionnaire-Revised. *Journal of Personality Assessment*, *96*(4), 445–458. https://doi.org/10.1080/00223891.2013.841171
